# K2R: Tinted de Bruijn graphs implementation for efficient read extraction from sequencing datasets

**DOI:** 10.1093/bioadv/vbaf111

**Published:** 2025-05-14

**Authors:** Léa Vandamme, Bastien Cazaux, Antoine Limasset

**Affiliations:** UMR9189 CRIStAL, Univ Lille, CNRS, Centrale, Lille F-59000, France; UMR9189 CRIStAL, Univ Lille, CNRS, Centrale, Lille F-59000, France; UMR9189 CRIStAL, Univ Lille, CNRS, Centrale, Lille F-59000, France

## Abstract

**Summary:**

Biological sequence analysis often relies on reference genomes, but producing accurate assemblies remains a challenge. As a result, de novo analysis directly from raw reads, without preprocessing, is frequently more practical. A common task across many applications is to identify reads containing a given *k*-mer in a dataset—essential for genotyping, profiling, compression, error correction, and assembly. While this resembles the well-studied colored de Bruijn graph problem, solving it at the read level is typically too resource-intensive. We show that this challenge becomes tractable by making realistic assumptions about genome sequencing datasets. To address it, we introduce Tinted de Bruijn graphs, a variation of the colored de Bruijn graph in which each read is treated as a unique source. We developed K2R, a scalable index implementing this model efficiently. We benchmark K2R’s performance (index size, memory usage, throughput, and construction time) against leading methods, including hashing techniques (Short Read Connector, Fulgor) and full-text indexing tools (Movi, Themisto). K2R successfully indexed two human datasets (T2T), handling up to 126X ONT coverage in under 9 hours with a peak of 61 GB RAM.

**Availability and implementation:**

Developed in C++, K2R is open source and available at http://github.com/LeaVandamme/K2R.

## 1 Introduction

The pursuit of direct and unbiased access to complete nucleic sequences remains a central focus in sequence analysis. Despite significant advancements over the past two decades, achieving accurate and comprehensive reference sequences continues to pose major challenges (He *et al.* 2023). A primary difficulty lies in the inherent complexity of assembly processes, both from a theoretical perspective (Nagarajan and Pop 2009) and in practical implementation (Nurk *et al.* 2022). These challenges are often exacerbated by the intrinsic properties of sequencing data, which can limit the direct applicability of assemblies to key biological questions, such as quantification or dataset comparison. Consequently, the *de novo* analysis of raw datasets holds paramount importance.

Since the introduction of the seed-and-extend paradigm (Altschul *et al.* 1990), numerous applications have heavily relied on identifying fixed-length word matches, commonly referred to as *k-mers*, to detect sequence similarities. A highly efficient representation of a *k*-mer set from sequencing datasets is the de Bruijn graph (Chikhi *et al.* 2015), which partially preserves the original ordering of *k*-mers. *K*-mer sets have found widespread applications across diverse domains, including quantification (Wang *et al.* 2012), dataset comparison (Ramos *et al.* 2024), phylogeny (Lyman *et al.* 2017), assembly (Bankevich *et al.* 2022), compression (Benoit *et al.* 2015), error correction (Limasset *et al.* 2020), and sequence alignment (Liu *et al.* 2016). The advent of short-read sequencing technologies has further highlighted the utility of the de Bruijn graph as a versatile and efficient structure for handling *k*-mers. Consequently, *k*-mer indexes have become well-researched data structures, with highly efficient implementations leveraging either hashing-based approaches (Marchet *et al.* 2021b; Pibiri 2022) or full-text indexing methods (Rossi *et al.* 2022, Alanko *et al.* 2023).

A significant limitation of such indexes is their inability to retain critical information, specifically the colocalization of *k*-mers within shared DNA fragments. In the context of short reads, this loss of information was considered negligible, as the *k*-mer size (*k*) typically approximated the size of the reads. However, for long reads, this limitation becomes far more impactful, as it results in the omission of highly valuable long-range information. One potential solution is the ability to identify the specific reads in which a given *k*-mer appears. Existing tools based on document scanning, while practical, have the drawback of requiring the entire, often redundant, dataset to be read for extracting the relevant reads, leading to very low throughput (Baire *et al.* 2024). In contrast, full-text indexes (Li 2014), which can efficiently locate patterns within indexed datasets, present a natural and more effective alternative. Recent advancements in BWT-based full-text indexes have led to the development of two novel structures that refine the previously dominant FM-index. The r-index (Bannai *et al.* 2020), a full-text index requiring space proportional to O(r), where *r* denotes the number of BWT runs for an input text of size *n*, introduces a suffix array sampling strategy that occupies only 2rlog(r) bits space. This innovation significantly reduces locate time from Ω(n/r) per occurrence (as in traditional FM-indexes) to O(log(n/r)). More recently, an extension of the r-index, br-index (Arakawa *et al.* 2022) (bi-directional index), has been developed enabling bidirectional extensions along the pattern search process. This use $O(r+r_{R})$ words of space, where $r_{R}$ is the number of BWT runs of the reversed text, and the locate time is O(occ), where *occ* is the number of occurrence of a pattern in the text. Movi (Zakeri *et al.* 2024), based on the move index (Nishimoto and Tabei 2021), achieves both O(r) space efficiency and O(1)-time queries. Nonetheless, these structures face challenges in compressing the high redundancy of reads, which often contain noise that disrupts redundancy and introduces irrelevant novel sequences to index.

This *k*-mer-to-reads problem closely resembles the well-studied colored de Bruijn graph challenge (Iqbal *et al.* 2012). A colored de Bruijn graph, constructed from a collection of documents, associates each *k*-mer with the list of documents in which it appears. In principle, each read could be treated as an individual document to leverage existing colored de Bruijn graph implementations. However, this approach becomes prohibitively expensive given the vast number of reads anticipated from gigabase-level genomes sequencing, easily reaching tens of millions when indexing 10 000 datasets is already very expensive (Marchet *et al.* 2021a, Marchet and Limasset 2023). Despite this, we emphasize that assumptions about the *k*-mer distribution in colored de Bruijn graphs are often limited. In contrast, we aim to demonstrate the problem tractability of indexing a sequencing dataset by leverage realistic properties.

In this work, we examine these properties and introduce practical techniques to leverage them effectively. For practical purposes, we introduce a scalable and efficient tool named K2R (*K*-mer to Reads), which leverages sequencing datasets properties, and perform an extensive benchmark against state-of-the-art structures capable of executing similar tasks. The K2R index, which can index a FASTA file of reads and then query one or more sequence files to accurately identify reads containing specified *k*-mers, all without yielding any false positives, is developed in C++, open source, and available on GitHub (http://github.com/LeaVandamme/K2R).

## 2 Methods

### 2.1 Notation and problem

In the following, all the strings will be defined in the DNA alphabet (an alphabet of size 4), but all the results could be generalized to a finite alphabet. A *k*-mer of a string *r* is a substring of *r* of length *k*. For a positive integer *k*, the *k-spectrum* of *r* is the set of all the *k*-mers of *r*. A canonical *k*-mer is represented by its lexicographically smallest form between the *k*-mer itself and its reverse complement. In this work, all *k*-mers are considered canonicals. For an integer m≤k and a specific total order of the set of the *m*-mers, the *m-minimizer* [or just minimizer (Roberts *et al.* 2004)] of a *k*-mer *x*, denoted by μ(x), is the smallest string of the *m-spectrum* of *x* according to a given order.

To define the set of similar reads $R^{\prime }\subseteq R$ (where *R* is the initial set of reads) to a sequence *s*, we choose to use the shared *k*-mers number as a filter mimicking (Solomon and Kingsford 2018). A read *r* will then be considered similar to *s* if the number of shared *k*-mers with *s* is greater than a threshold *t*, i.e. |k-spectrum(r)∩k-spectrum(s)|≥t.

### 2.2 Tinted de Bruijn graph

To support multiple queries on the same set of reads, we need to store, for each *k*-mer *x*, the set of reads where it appears, i.e. {r∈R:x∈k-spectrum(r)}. As a de Bruijn graph can be viewed through a set of *k*-mers [by storing arcs, or by testing 4 extensions (alphabet size) to access to possible successive *k*-mers], these two notions are commonly conflated.

Given a document collection F (sequencing datasets or assembled genomes), which is a cover for the sets of all reads *R* ($\cup_{F\in F}F=R$) and implies that the union of these documents represents a set of reads, a *colored de Bruijn graph* is defined as a de Bruijn graph where each *k*-mer is associated to the documents that contain it, i.e. a mapping from $\cup_{r\in R}k-\text{spectrum}(r)$ to P(F). F is the set of the fasta files, and each *k*-mer is associated with the list of fasta files where it appears. Similar to the colored de Bruijn Graph, we introduce the *Tinted de Bruijn graph* which is a de Bruijn graph where each *k*-mer is associated to the reads that contain it, i.e. a mapping from $\cup_{r\in R}k-\text{spectrum}(r)$ to P(R). The Tinted de Bruijn graph can be seen as a special case of colored de Bruijn graph where each read from a sequencing dataset is seen as a distinct source.

We first remark that such structure could, to some extent, recover its original dataset by navigating the de Bruijn graph and reconstructing the reads. However, in the case of duplicated region within a given read, we can obtain several possible distinct “assemblies” of such reads (multiple Eulerian paths in the induced subgraph of the de Bruijn graph by these reads). This means that the Tinted de Bruijn graph is not reversible (impossibility to turn up the initial reads and thus some information is lost). Since the presence of repeated *k*-mers corresponds to these nonunique assemblies, by storing, in extra space, the different positions in each read of these *k*-mers, we obtain a reversible version of the *Tinted de Bruijn graph*, which we call the *Reversible Tinted de Bruijn graph*. This notion has already been implemented in Counting de Bruijn graphs Karasikov *et al.* (2022), with the use of “*k*-mer coordinates,” representing the occurrence positions of a certain *k*-mer in the input stream, enabling reconstruction of the original sequences. This reversibility property makes this Reversible Tinted de Bruijn Graph an interesting new element of compressed full-text data structure family, as BWT and FM-index.

Instead of storing the Tinted de Bruijn graph which can be costly, we focus in this work on a lossy version of the Tinted de Bruijn graph, called *Minimizer Tinted de Bruijn graph*, where minimizers are used to represent the *k*-mers as each minimizer *y* is associated with the reads that contains a *k*-mer that has *y* as minimizer, i.e. {r∈R:y∈{μ(x):x∈k-spectrum(r)}}.

**Figure 1.**
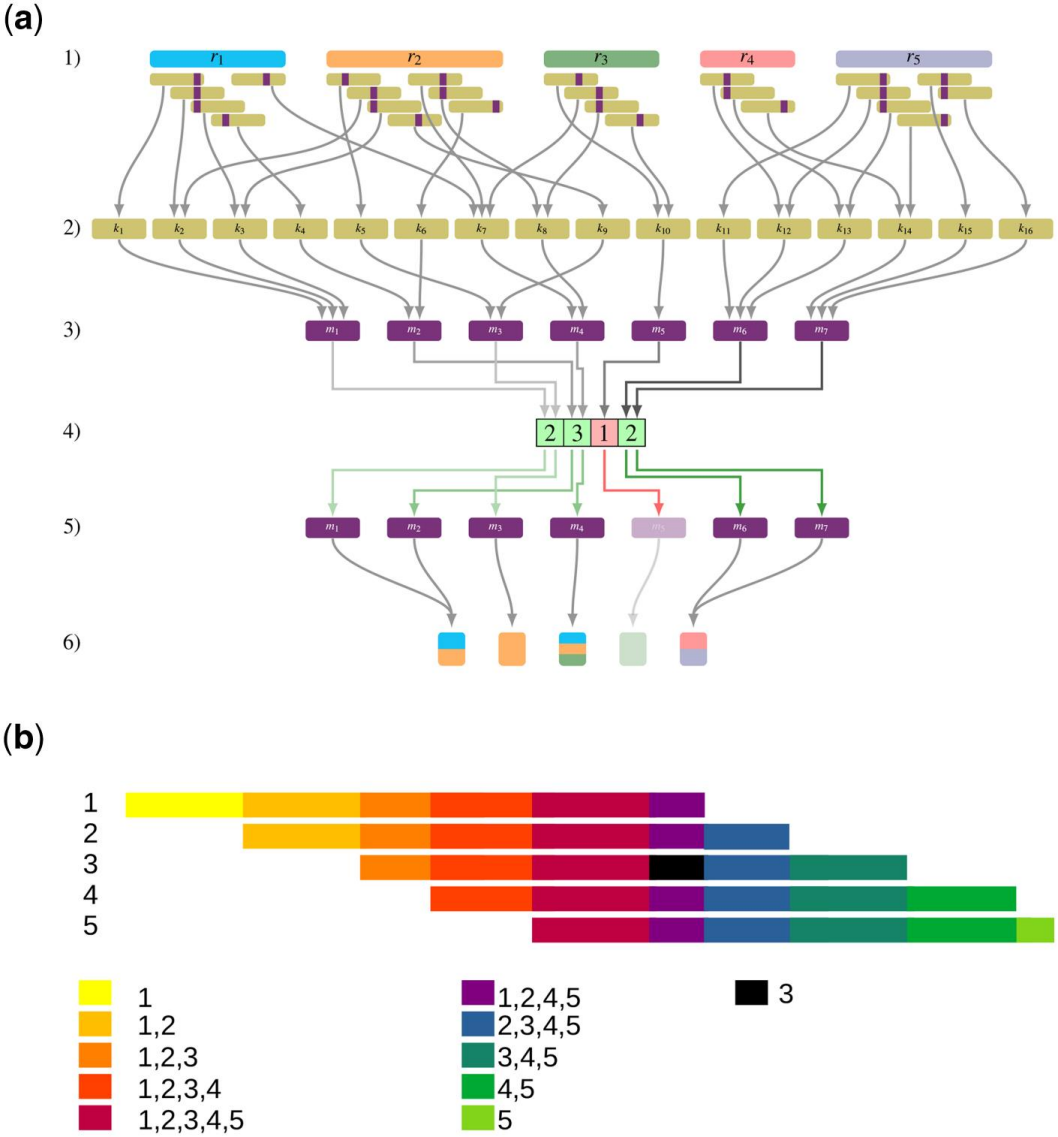
(a) An example of our lossy version of the Tinted de Bruijn Graph. For a set of reads (1), we can extract the set of *k*-mers (2). Each *k*-mer (2) is linked to a minimizer (3) with possible collisions. By using counting filter (4), we can remove weak minimizer (5). Each remaining minimizer (5) is linked to a color (6). (b) A simple example of how overlapping reads create subsequences whose *k*-mers are the same colors. We also display the impact of a sequencing errors in the black region that creates read specific sequences.

As for all *k*-mer *x*, {r∈R:x∈k-spectrum(r)}⊆{r∈R:μ(x)∈{μ(z):z∈k-spectrum(r)}}, the Minimizer Tinted de Bruijn graph can have false positives but no false negatives. In addition, the scattered positions of the minimizers and the minimizers shared between different *k*-mers mean that this structure is not reversible. In the following, we present our efficient implementation of the Minimizer Tinted de Bruijn graph dubbed K2R (see Fig. 1).

### 2.3 Overview

Given the conceptual similarity between the Tinted de Bruijn graph and the Colored de Bruijn graph, a naive implementation of a Tinted de Bruijn graph would involve an associative structure, such as a hashmap, linking *k*-mers to the list of read identifiers that contain them. Interestingly, an initial tool, SRC (Marchet *et al.* 2020), developed for short reads, actually implements this strategy using an efficient Minimal Perfect Hash Function (MPHF) (Limasset *et al.* 2017) as the associative structure. However, we argue that the cost of such a structure would be prohibitively high for large datasets. For example, a human long-reads dataset of 10 million 10 kb reads (approximately, 33X coverage) would represent more than 3 billion *k*-mers requiring 24 GB of storage (for k=31), and each *k*-mer would be associated with dozens of read IDs, necessitating hundred bytes per association. This would mean several hundred gigabytes of storage without any overhead and without accounting for sequencing errors. In this section, we describe the assumptions we can make when indexing a sequencing dataset and how we can exploit such properties to optimize this naive solution.

### 2.4 Sparse signal

The primary challenge in scaling colored de Bruijn graphs arises from the inherent difficulty of making a priori assumptions about the *k*-mer distribution within a dataset collection (Almodaresi *et al.* 2019). In contrast, sequencing datasets predominantly target sequences significantly larger than a kilobase, with relatively low coverage that rarely exceeds a hundred-fold. This coverage expectation implies that most *k*-mers will be present in a number of reads closely matching the coverage level, marking their presence in an exceptionally small fraction (by several orders of magnitude) of the total reads, as even bacterial genome sequencing can yield millions of reads. Consequently, unlike with colored de Bruijn graphs, it becomes viable to employ sparse encoding rather than bitvector representations to efficiently denote the presence of *k*-mers in reads.

Employing a 32-bit identifier list to represent *k*-mers turns out to be prohibitively expensive in terms of memory usage, demanding hundreds of bytes per *k*-mer for a standard whole-genome sequencing dataset. A more efficient alternative involves using a sorted list with delta encoding, which significantly reduces the memory footprint. Initially, one might assume that applying compression techniques at such a scale could detrimentally affect construction or query times. However, advancements in compression technology have led to the development of highly optimized algorithms (Trotman and Lin 2016), such as TurboPFor Heinzl *et al.* (2021), used in this work. These algorithms leverage SIMD (Single Instruction, Multiple Data) instructions, enabling near-state-of-the-art compression efficiency with minimal impact on overall throughput.

As an example, for a HiFi *E. coli* sequencing with 200X coverage and an error rate of 1%, the integer lists typically goes from a size of 600 bytes to a size of 200 bytes, a three-fold improvements, without perceptible wall-clock drawback.

### 2.5 Redundant colors

An important observation in sequencing datasets is the considerable proportion of overlap among reads, which is directly related to the level of coverage. For instance, with 10X coverage, one can anticipate that reads will overlap by approximately 90% of their length, while at 100X coverage, the overlap can increase to 99%, and so forth. In this context, colors in our dataset predominantly represent combinations of reads that overlap, covering specific genomic regions, as illustrated in Fig. 1. Consequently, the diversity of distinct colors observed is relatively limited, since each new read starting position (and consequently, each end position) generates a novel combination. Assuming an ideal scenario devoid of sequencing errors, the number of distinct colors would be expected to increase linearly with the number of reads, necessitating approximately O(log(N)) bits to encode colors for *N* reads. However, the scenario becomes more complex with the presence of *k*-mers repeated across the genome or *k*-mers that contain sequencing errors. Sequencing errors introduce a significant number of additional colors in two ways: firstly, by creating singleton colors unique to individual reads, and secondly, by generating “incomplete” colors that resemble existing colors but with “gaps” due to the absence of one or more reads, attributable to missing *k*-mers caused by sequencing errors. This leads to the observation that a substantial number of *k*-mers actually share the same color, a property determined by the coverage, read length, and sequencing error rate.

To leverage this characteristic efficiently, we have developed a strategy that indexes only distinct colors, similar to Rainbowfish (Almodaresi *et al.* 2017), by associating them with unique color identifiers, thereby avoiding the redundancy of storing numerous identical colors. This approach entails maintaining a table that links each *k*-mer to its corresponding color identifier and another table that maps each identifier to the actual list of read identifiers. We chose an efficient stored HashMap utilizing Robin Hood backward shift deletion, available at https://github.com/martinus/unordered_dense, which allows us to optimize both memory usage and computation time. This method enables the storage of each distinct list precisely once, albeit at the cost of a minor overhead due to the inclusion of identifier integers.

### 2.6 Successive *k*-mers

Upon closer inspection, we can observe more specifically that overlapping *k*-mers from sequence reads are frequently assigned to the same color, primarily because they originate from identical genomic regions and are thus present in the same reads (see Fig. 1). Theoretically, one could attempt to reconstruct such regions and map *k*-mers to their respective regions, which would reflect their shared colors; however, this process is anticipated to be nearly as resource-intensive as genome assembly.

An alternative, more dynamic approach to leverage this characteristic involves the utilization of minimizers. Minimizers, which are the minimal *m*-mers within *k*-mers, are often shared among overlapping *k*-mers (Pibiri *et al.* 2023), leading to a significantly reduced number of unique minimizers compared to the total number of *k*-mers in a dataset. Ideally, a scheme would select a unique minimizer for every group of k−m  *k*-mers. Through the application of “random minimizers,” which employ a hashing function, it is estimated that the number of selected minimizers required is twice the minimal theoretical number (Groot Koerkamp and Pibiri 2024). Nevertheless, by adopting advanced minimizer selection algorithms (Zheng *et al.* 2020, Pellow *et al.* 2023, Groot Koerkamp and Pibiri 2024), one can surpass these expectations and further reduce the number of selected minimizers in practice.

However, a notable limitation of this technique is the inherent association of all *k*-mers sharing a minimizer with the same color, potentially leading to false positives. Each minimizer is linked to every read in which it appears, and having *n* shared minimizers is a necessary condition for obtaining *n* shared *k*-mers, thereby preventing false negatives. This methodology may result in distinct *k*-mers, which occur in separate reads without shared *k*-mers, logically possessing distinct colors, being assigned the same minimizer and consequently, the same color. Such scenarios, albeit infrequent, could inadvertently introduce false positives, adversely affecting downstream analyses. Conversely, this propensity for false positives may inadvertently counteract the generation of “subcolors” due to sequencing errors, as previously discussed, by incorrectly assigning some erroneous *k*-mers to the correct color, where the genuine genomic *k*-mer would be assigned. We posit that these false positives can be efficiently managed by calculating and reporting the actual number of shared *k*-mers when the selected reads are analyzed, thus providing a balanced approach to minimizing data misinterpretation while maximizing scalability.

### 2.7 Handling sequencing errors

A fundamental obstacle in addressing our problem is the occurrence of sequencing errors, which introduce novel sequences (and *k*-mers). These are predominantly unique, rendering them incompressible and biologically irrelevant. Moreover, these errors dilute the overall redundancy by transforming abundant sequences into rare novel variants. Traditional full-text indexing approaches often struggle in these scenarios, as they are designed to index entire texts indiscriminately and are typically employed on assembled sequences “cleaned” of sequencing errors for this reason.

In contrast, the success of the de Bruijn graph approach can be partly attributed to its ability to manage sequencing errors effectively by leveraging the concept of *k*-mers abundance. In practice, *k*-mers containing sequencing errors are expected to occur infrequently, whereas the abundance of genomic *k*-mers is closely aligned with sequencing coverage. Thus, implementing a simple abundance filter that retains “solid” *k*-mers appearing more than *T* times in the dataset and excluding “weak” *k*-mers that appear fewer than *T* times can eliminate nearly all erroneous *k*-mers with minimal loss of relevant *k*-mers, provided the threshold *T* is optimally set according to the observed abundance. However, this type of filter, by removing certain *k*-mers, may lead to false negatives. Indeed, we can no longer distinguish between absent *k*-mers and those that are either too rare or too frequent and thus filtered out. In practice, these filtered *k*-mers are generally not relevant to retain.

Since we focus on indexing minimizers, we introduce a novel variation of this filtering technique applied at the minimizer level. It follows logically that if a *k*-mer is solid (seen more than *T* times), then its minimizer is also considered solid. Consequently, excluding weak minimizers does not result in the exclusion of solid *k*-mers. However, minimizer-level filtering may not effectively eliminate erroneous *k*-mers that share a minimizer with solid *k*-mers. This leads us to anticipate that minimizer filtering is less precise than *k*-mer filtering, with the benefit of significantly reducing the inherent counting cost, as it involves quantifying an order of magnitude fewer keys.

## 3 Results

All experiments were conducted on a single cluster node equipped with an Intel(R) Xeon(R) Gold 6130 CPU @ 2.10 GHz, 128GB of RAM, and running Ubuntu 22.04. To validate our results on real data, we chose to perform the same analysis on closely related *E. coli* sequencings. Specifically, we selected a recent ONT sequencing with a low error rate, approximately 3% (Accession SRR26899125), a 20 kb HiFi sequencing (Accession SRR11434954), and a HiSeq X Ten paired-end sequencing (Accession DRR395239). To extend our evaluation to a medium-sized genome, we included two *C. elegans* datasets, one ONT sequencing (Accession SRR24201716), and another HiSeq X Ten paired-end sequencing (Accession ERR10914908). To complete, we also used a metagenomic dataset: a 17 Gb HiFi Zymo Biomics community sequencing, containing 21 strains of 17 species (Accession SRR13128014), already used by assemblers like metaMDBG (Benoit *et al.* 2024). Finally, to assess K2R scalability, we selected two human datasets from the T2T project. The HiFi datasets (SRX7897685, SRX7897686, SRX7897687, SRX7897688, and SRX5633451) together amount to 56.8X coverage, comprising both 20 kb and 10 kb libraries. The ONT sequencings, detailed at http://github.com/marbl/CHM13/blob/master/Sequencing_data.md, total 126X coverage with an approximate error rate of 6%.

### 3.1 State of the art

We categorize tools capable of processing Tinted de Bruijn graph queries into two main types. The first type includes full-text indexes, which can locate *k*-mer occurrences within an indexed read file. Utilizing a starting position array enables the determination of specific reads in which a queried *k*-mer is present. The second approach involves *k*-mer indexes, which rely on associative structures such as hash functions. Despite significant advancements in reducing the memory footprint of *k*-mer indexing to below 10 bits per *k*-mer (Pibiri 2022), a major challenge persists in managing the associated read identifier lists, as these lists are expected to be larger by several orders of magnitude.

Recent developments in BWT-based full-text indexes have introduced several innovative structures enhancing the capabilities of the FM index, which dominated the field for over a decade. The latest tool utilizing the r-index for large-scale queries is SPUmoni (Ahmed *et al.* 2023), while br-index (Arakawa *et al.* 2022) is an extension of the r-index and full-br-index (Arakawa *et al.* 2022) a fully functional br-index. Movi (Zakeri *et al.* 2024) is the sole tool based on the move index. Given the efficiency of the move index, particularly in query time (O(1)), we opted to include Movi in our benchmark over SPUmoni.

To our knowledge, the only specialized tools implementing *k*-mer to reads index is SRC (Marchet *et al.* 2020) (v1.2.0) employing Minimal Perfect Hash Function (Limasset *et al.* 2017), but we also included Themisto (Alanko *et al.* 2023) (v3.2.2) based on a Spectral BWT, Movi (Zakeri *et al.* 2024) based on the move index, Fulgor (Fan *et al.* 2024) (v3.0.0) based on Minimal Perfect Hash Functions (Pibiri and Trani 2021), LueVari (Alipanahi *et al.* 2021) based on the BWT-like structure BOSS (Bowe *et al.* 2012), and full-br-index (Arakawa *et al.* 2022) in our benchmark.

As outlined in the introduction, a fundamental limitation of both full-text and *k*-mer-based indexes is their difficulty in handling sequencing errors, leading to the indexing of irrelevant sequences. In contrast, *k*-mer abundance filtering enables *k*-mer indexing methods to effectively exclude nonessential *k*-mers by considering their frequency of occurrence.

In our benchmarking, both SRC and K2R indexed 31-mers while excluding those that appeared only once or more than 5000 times in the dataset. This creates a theoretical disparity in comparisons, as full-text indexes inherently index the entire text, and Fulgor and Themisto do not offer a similar option. However, in practical terms, users of these techniques have no other options but to employ them as they currently exist, and most analyses actually filter out at least unique *k*-mers as they are considered unreliable. All minimizers-based methods in our benchmark use minimizers of length 15.

We finally decided to omit full-br-index from the benchmark since the smallest dataset provided (10X coverage of simulated reads from the *E. coli* genome) took more than 24 minutes to process (about 20 times longer than Movi, 150 times longer than K2R). This suggests that full-br-index does not scale well for this application. Similarly, LueVari, which reports results (44 minutes and 8.7 GB of RAM for 2 898 708 reads of length 100) indicating that it will not scale within the scope of our benchmark, was not included as we were unable to compile it successfully.

### 3.2 Index construction

We first evaluate the cost of constructing an index from whole genome sequencing datasets with the different tools.

**Figure 2.**
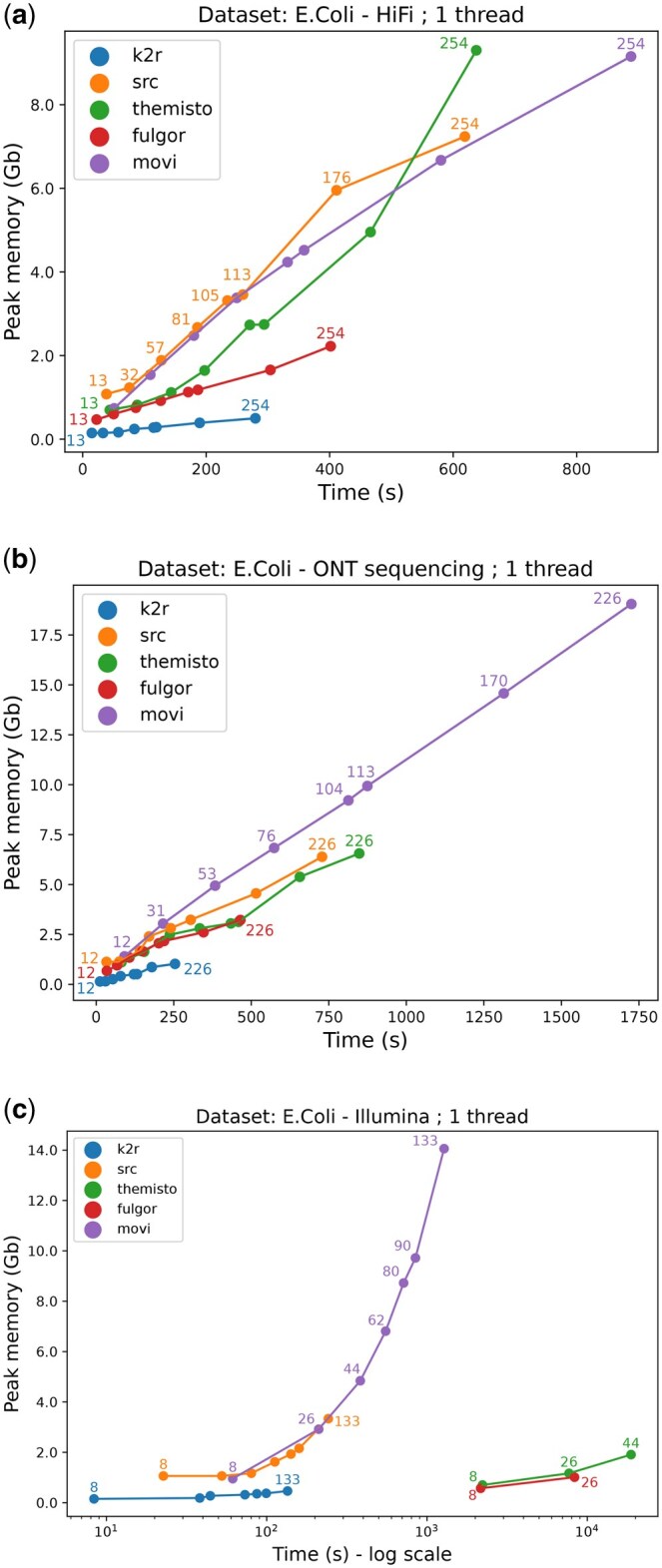
Memory peak and wall-clock time used during the index construction with three different kinds of data with varying coverages (specified on the labels) from *E. coli* genome: HiFi (a), ONT (b), and Illumina (c), where we notice that both Themisto and Fulgor cannot scale to a 62X and 44X coverage, respectively. This last graph is in logarithmic scale for readability purposes.

In Fig. 2, we assess the time and memory required according to the input coverage on three *E. coli* datasets. We observe that for all tools, both time and memory requirements grow linearly with the input size. However, their respective performance varies greatly.

Overall, we observe that K2R is significantly faster and memory efficient than the state of the art, while Movi is slower and more memory expensive. This effect is quite small for HiFi data but is amplified with ONT data. For Illumina data, we note that the time required by Fulgor and Themisto increases dramatically. This is due to their operational mode, which requires a unique sequence per file. In our case, this means one file per read, leading to numerous disk accesses and a corresponding performance bottleneck.

**Figure 3.**
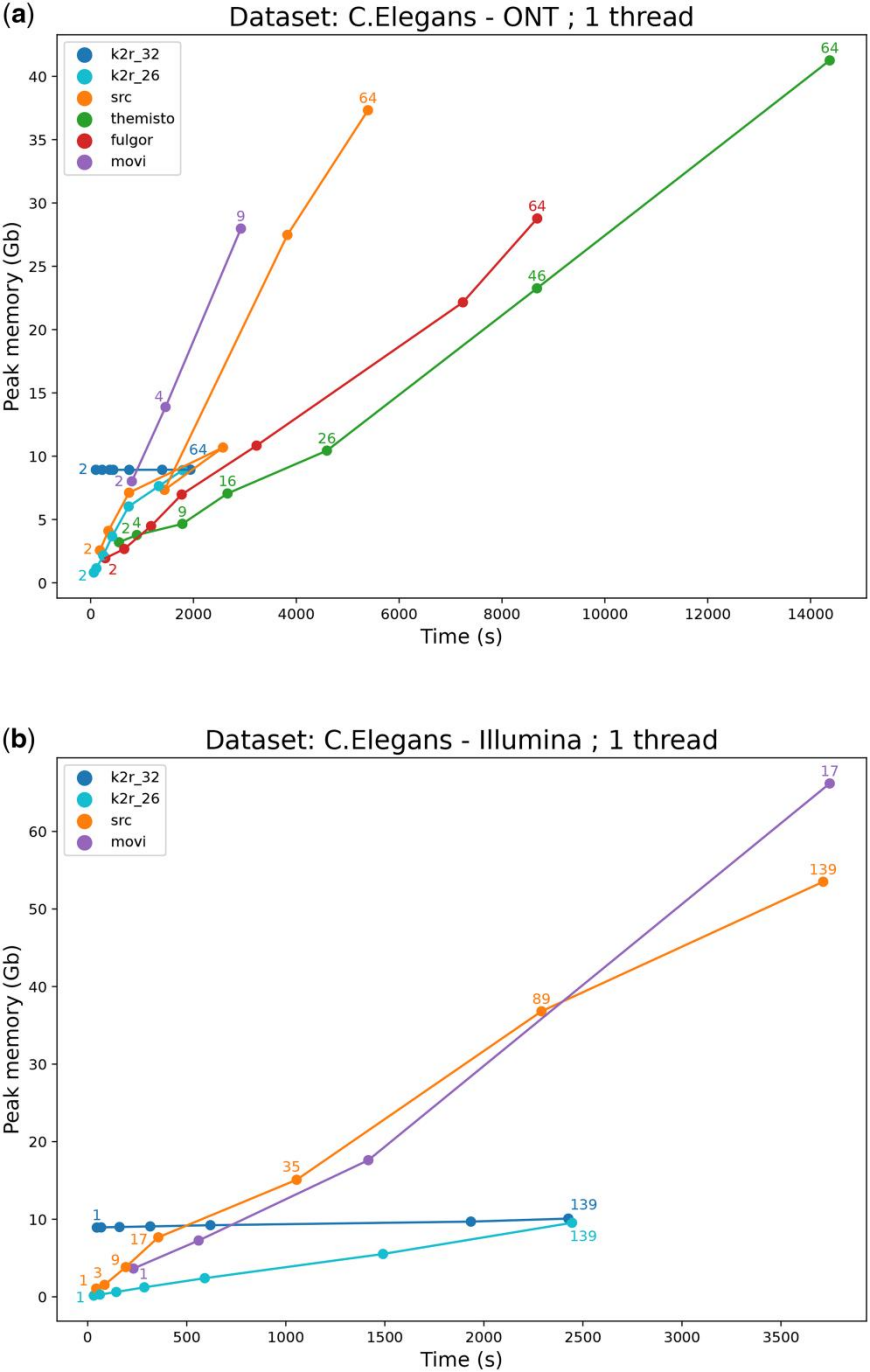
Memory peak and wall-clock time used during the index construction with two different kinds of dataset from *C. elegans* genome with varying coverages (specified on the labels): ONT (a), we notice that Movi cannot scale up for a coverage greater than 9X. Illumina (b), we notice that Movi cannot scale up for a coverage greater than 17X. Themisto and Fulgor cannot be used with this type of data because of their mode of use. K2R 26 and K2R 32 refer to the counting bloom filter size ($2^{26}$ or $2^{32}$) used to index.

We performed the same experiment on the *C. elegans* genome, which is 20 times larger, as shown in Fig. 3 and obtained similar results, highlighting K2R’s scalability in memory and, more importantly, in time. Since most of the memory is allocated to filter minimizers in a fixed size counting bloom filter ($2^{32}$) across experiments, this explains K2R’s almost constant memory, even for larger datasets. For comparison, we included a curve using a smaller filter size ($2^{26}$), which leads to more minimizer collisions and potentially a higher rate of false positives in the structure. However, Fig. S1 in the Appendix shows that these false positives have minimal impact, as the true positive rate remains above 90% on average with a threshold of 0.3. We notice that Themisto and Fulgor does not appear for Illumina dataset. This is because these tools require a unique sequence per file, meaning that in our case, one file is created per read. These tools are better suited for indexing entire genomes, not datasets divided into individual reads.

As a validation, we also performed the benchmark on simulated reads from the reference genomes, obtaining very similar results displayed in Fig. S3 of the Appendix. The analysis was also conducted using multiple threads, as shown in Figs S7 and S8 of the Appendix.

Finally, we applied this analysis to the two human datasets, using K2R exclusively due to scalability issues with other tools. We tested two values for maximum abundance (256 and 1000). We note that this restriction of 256 is common and used by assembly tools like MECAT (Xiao *et al.* 2017) or aligners like BELLA (Guidi *et al.*), skipping *k*-mers seen more than 128 times. The results, presented in Table 1, reveal the high cost of indexing highly repeated *k*-mers in complex genomes like that of humans. Including such *k*-mers nearly doubles both time and memory requirements. These results demonstrate that such indexes are quite tractable to construct on various datasets, even on gigabase-scale genomes, on a modest workstation.

**Table 1.** Index construction results on two *Human* datasets (56X HiFi and 126X ONT) using K2R, depending on the maximum abundance of minimizers.^a^

Abundance	Max abundance: 256		Max abundance: 1000	
Dataset	HiFi 56X	ONT 126X	HiFi 56X	ONT 126X
RAM	16 GB	30 GB	39 GB	61 GB
Wall-clock time	2h37	5h53	4h30	9h05
Structure size	7.6 GB	10.71 GB	20.5 GB	30.1 GB

a For each value, the RAM used, the wall-clock time, and the structure size are presented in gigabytes and hours.

As a metagenomic experiment, we indexed the 17 Gb Zymo dataset. For the same reason as the Human datasets, we used K2R exclusively due to scalability issues. It only took 8.9 Gb of RAM and 32 minutes. The index size is 556 Mb.

To further analyze the impact of noise, we evaluated the time and memory requirements according to the input error rate, with coverage fixed at 50X. The results are presented in Fig. S4 in the Appendix. Since testing such parameters on real data poses challenges, this benchmark was conducted exclusively on simulated reads from the *E. coli* and *C. elegans* reference genomes. For the *E. coli* data, this analysis revealed two distinct patterns. The error rate had minimal impact on the construction time for K2R and SRC, while it affected Fulgor, Movi, and Themisto. In the case of *C. elegans*, the error rate has a minor influence on the memory usage of K2R and Movi (though Movi could not scale beyond an error rate of 0.5%). It can even decrease with a higher error rate as fewer *k*-mers are indexed due to abundance filtering. Full-text indexes, on the other hand, were significantly hindered by the error rate. They performed well with a low error rate but became much more costly with higher error rates. If SRC outperforms other methods in terms of time, regardless of the error rate, K2R itself is vastly superior in both terms of time and memory efficiency.

### 3.3 Index size

**Figure 4.**
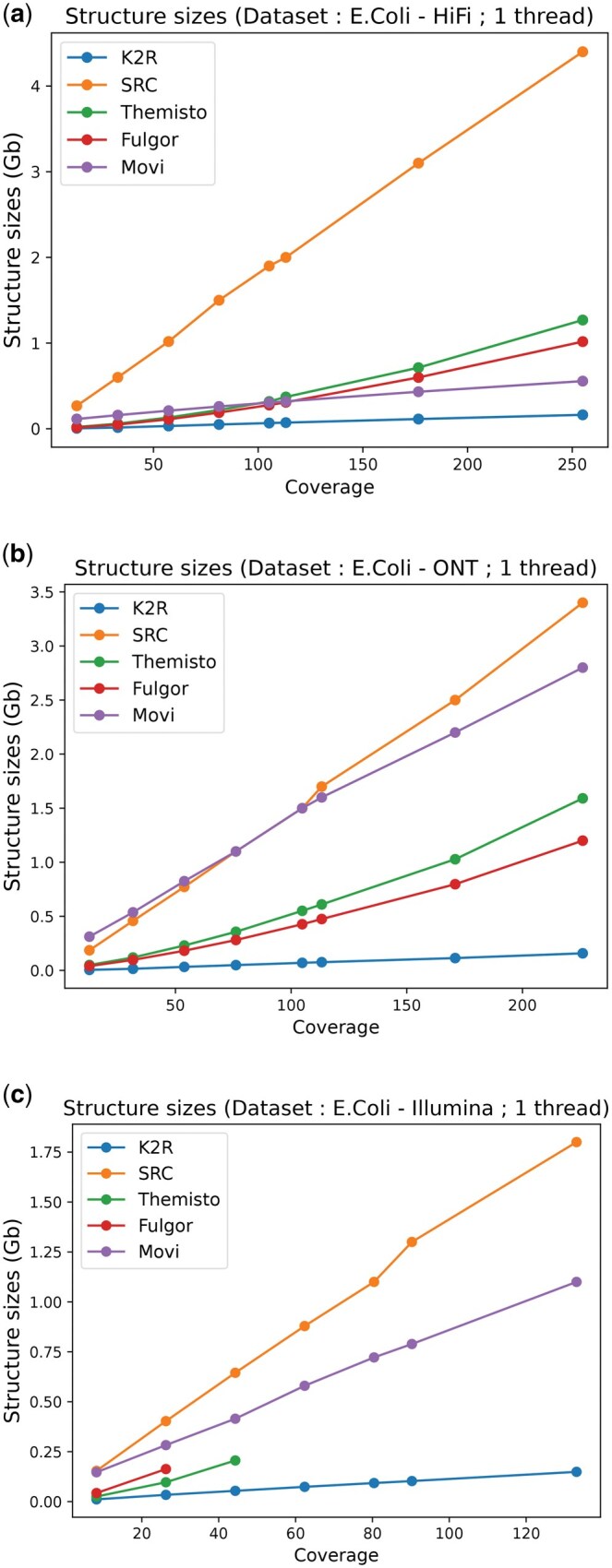
Comparison of the index size, for three different *E. coli* genome datasets according to the input coverage: (a) HiFi dataset (top), (b) ONT dataset (center), and (c) Illumina dataset (bottom), where we notice that Themisto and Fulgor cannot scale up to coverages greater than 44 and 26, respectively.

**Figure 5.**
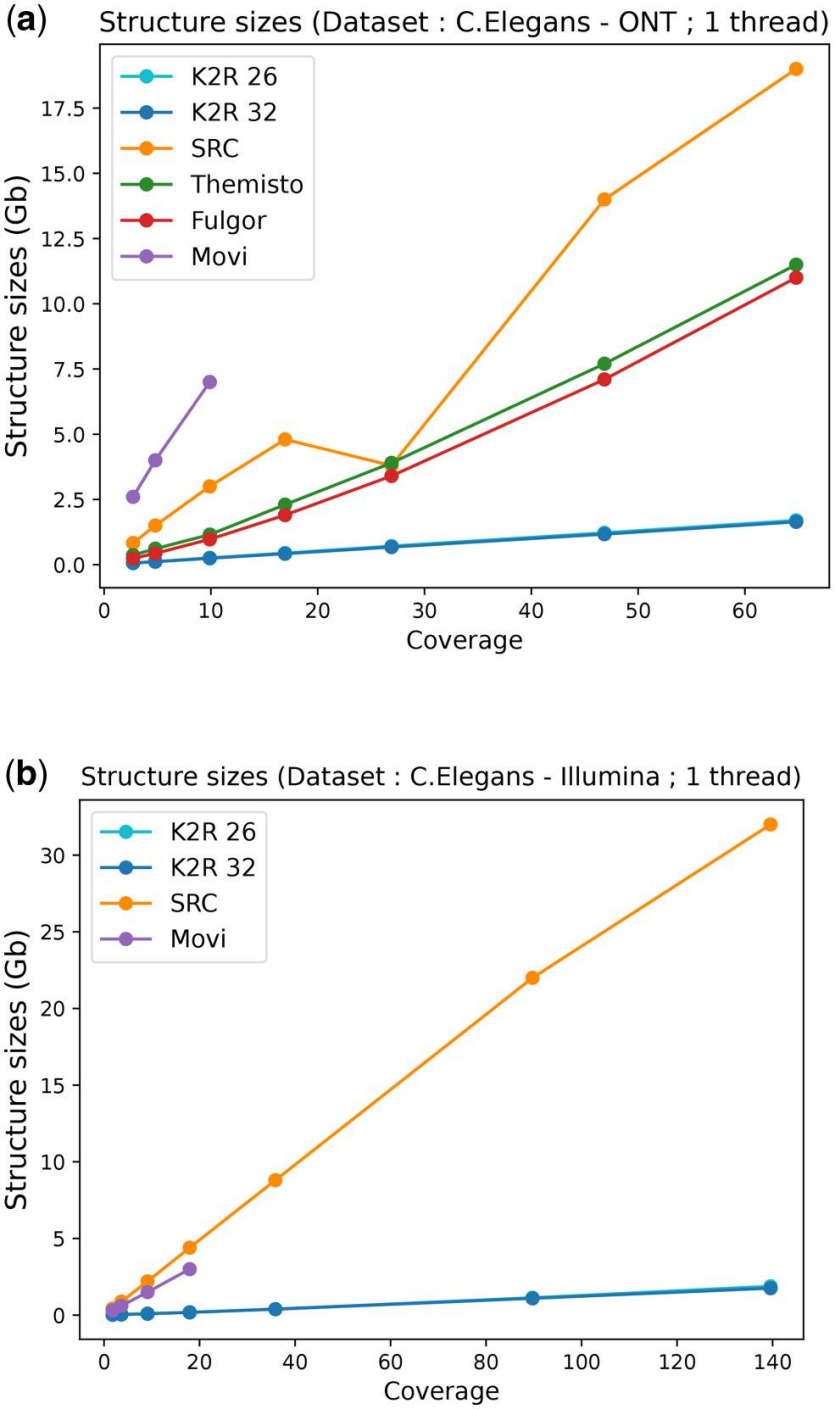
Comparison of the index size, for two types of datasets from *C. elegans* genome according to the input coverages: (a) ONT and (b) Illumina. K2R 26 and K2R 32 refer to the counting bloom filter size ($2^{26}$ or $2^{32}$) used to index; here, the two curves are merged because the difference is almost nonexistent.

While the construction cost is a key factor in tractability, the actual final index size is more important since the construction needs to be performed only once but the index can be used multiple times. As before, we first assess the impact of coverage in Fig. 4 for the *E. coli* dataset and in Fig. 5 for the *C. elegans* datasets.

We observe distinct patterns across the different data types. For HiFi data, all tools, except SRC, perform quite well using less or about a gigabyte for 200X coverage. However, for ONT data, only K2R maintains this efficiency, and Movi grows similarly to SRC. Illumina data follow the same trend but due to their usage mode, Themisto and Fulgor cannot be tested at high coverage levels. The differences between SRC/Movi and K2R are even more pronounced for the *C. elegans* dataset. Notably, varying the counting Bloom filter size in K2R between $2^{32}$ and $2^{26}$ has minimal impact on the final structure size, as collisions remain negligible. To validate these results, we benchmarked simulated reads from the *E. coli* and *C. elegans* genomes, obtaining comparable outcomes (see Fig. S5 in the Appendix). Finally, as a scalability experiment, we report the index size from human datasets (skipping *k*-mers seen more than 1000 times). While indexing high-coverage ONT human datasets is relatively inexpensive in practice (about 30GB, which is more than an order of magnitude smaller than the actual dataset: 381GB), we find that the HiFi data index is even an order of magnitude smaller (20.5GB instead of 165GB), indicating that sequencing errors can significantly impact the size of such indexes (see Table 1).

Following the experimental plan, in a second experiment, we evaluate the impact of sequencing error rates on index size using simulated data, as presented in Fig. S6 in the Appendix. As expected, a higher error rate generally means a larger index file. An exception is observed with SRC, where increased error rates reduce the number of colors indexed, leading to a smaller overall index size. Apart from this effect, K2R is almost always an order of magnitude smaller than its competitors, especially for larger datasets.

### 3.4 Queries

We now assess the query throughput of the different indexes. Instead of measuring the query time for a single query (which is challenging due to overheads such as index loading), we tested batches of sequences ranging from 1000 to 10 000, each between 500 and 10 000 bases long. We conducted two types of queries: positive queries, where reads were extracted from the *E. coli* reference genome, and negative queries, where reads were randomly generated. All queries were executed using 24 threads. The results are detailed in Figs S8 and S9 of the Appendix. It is important to note that Movi was excluded from these results, as it performs a different type of operation, focusing on the presence/absence and similarity rather than returning the location of reads.

First, concerning the memory peak, K2R, Themisto and Fulgor are constant, while SRC grows quickly with the number of queries. This trend is consistent for both positive and negative queries. In terms of time, however, we have different observations. SRC is still the less efficient (at least an order of magnitude slower than the others, two for 10 000 queries). Themisto follows, performing similarly to K2R for positive queries (around 10 s per experiment) but slightly slower for negative queries. K2R ranks next, with Fulgor emerging as the fastest, particularly for negative queries, and only marginally slower for a large number of positive queries. In practice, the time difference between K2R, Themisto, and Fulgor is minimal (less than 10 s in all cases), rendering all three tools, including K2R, highly efficient.

We also measured, as part of these queries, the false positive rate that can be observed for two different error rates (Fig. S1 of the Appendix) and several minimizer sizes (Fig. S2). For an error rate of 1%, we observe a false positive rate below 10%. The minimizer size also plays a role, as expected, since a larger minimizer size makes each minimizer less frequent, thereby reducing collisions. Notably, the false positive rate decreases from over 40% for minimizers of size 11 to less than 25% for a size of 21.

In summary, our analysis leads us to three key conclusions. Firstly, the K2R index stands out as the most efficient for construction, significantly reducing memory and time consumption, in stark contrast to the resource-intensive nature of BWT-based indexes. Secondly, and perhaps most critically, K2R indexes are markedly smaller than current leading tools, surpassing them by several orders of magnitude, independently of coverage and error rates. Thirdly, queries processed through K2R are fast and as memory frugal as Themisto and Fulgor. These findings display the practical feasibility of employing Tinted de Bruijn graph methodologies at scale, highlighting the effectiveness of the presented approaches.

## 4 Discussion

The K2R index holds significant potential for diverse applications requiring scalable read-level resolution, such as metagenomics or transcriptomics quantification, assembly, or genotyping. Future work will aim to integrate and fine-tune it for these targeted applications.

Besides developing applications, there are multiple ways to improve our proposed implementation to make it more scalable and efficient. A trivial enhancement would be the use of static, memory-efficient associative structures, which could present an interesting trade-off for index construction efficiency. We propose to exploit two main characteristics of whole genome sequencing datasets: the existence of an underlying sequence and, therefore, overlapping reads, and the relatively uniform redundancy and error rates. To significantly improve performance beyond exploiting super-*k*-mers, a static index could infer a good ordering (similar to the genome-induced ordering) based on some form of assembly. Using this ordering, we could exploit the fact that many successive *k*-mers in this pseudo-genome would be associated with the same data, improving both query time and index size by reducing redundancy. Furthermore, when not identical, successive *k*-mers often share very similar patterns (see Fig. 1). Therefore, colors themselves could also be encoded relative to the previous colors, as we expect most modifications between two successive colors in the genome to involve the addition or removal of a read. Various degrees of such delta encoding could offer efficient time/memory trade-offs.

Optimizing the ordering of reads in sequencing datasets offers a promising approach to significantly enhance the compressibility of list encodings. By reordering the reads in the file to group similar/overlapping reads together, their shared *k*-mers would be associated with highly compressed lists. Such read ordering could be based on clustering, overlap detection steps, or alignment to the aforementioned pseudo-genome. As a proof of concept, we compared the index size constructed from simulated reads in random order and ordered by their origin position in the genome. For a 200X coverage of *C. elegans* with a 1% error rate, the color index size reduced from 3.7GB to 1.3GB, a three-fold improvement. With a 0.1% error rate, the color index size goes from 3.4GB to 251MB, an order of magnitude smaller. This displays the interest of optimizing read ordering to facilitate their indexability or compression, a problem that starts to regain interest for long reads datasets (Lee and Song 2022).

Even though the Minimizer Tinted de Bruijn graph is extremely lightweight and could fit many uses, an actual (*k*-mer) Tinted de Bruijn graph could be interesting for applications where actual de Bruijn graph navigation is required or when a very low number of *k*-mers are queried, and every false positive could be critical. Such an index could mimic the K2R representation and rely on efficient *k*-mer indexes like SSHash (Pibiri 2022).

Additionally, in scenarios where there is a need to search for sequences across multiple datasets, the ability to merge two indexes would be highly beneficial.

Beyond these direct considerations, we believe that the Tinted de Bruijn graph structure is an intriguing representation of a sequencing dataset. Regular de Bruijn graphs are a robust representation of short-read datasets because they encapsulate most of their information in a lightweight and scalable manner. In this manuscript, we demonstrated that a lossy practical implementation is quite lightweight and scalable while retaining almost all the useful information from the original dataset. As a result, while the de Bruijn graph has been deemed unsuitable for noisy long reads, we argue that the Tinted de Bruijn graph is the right representation for such datasets.

We also introduced the concept of the Reversible Tinted de Bruijn graph as a novel form of compressed full-text index adapted to sequencing data. Since we only need to manage duplicated *k*-mers within a given read, we believe that the additional cost to allow a Tinted de Bruijn graph to be reversible will be quite low in most scenarios. The theoretical and practical study of such a structure will be the natural continuation of this work.

## Supplementary Material

vbaf111_Supplementary_Data

## References

[vbaf111-B1] Ahmed OY , Rossi M, Gagie T et al Spumoni 2: improved classification using a pangenome index of minimizer digests. Genome Biol 2023;24:122. 37202771 10.1186/s13059-023-02958-1 PMC10197461

[vbaf111-B2] Alanko JN , Vuohtoniemi J, Mäklin T et al Themisto: a scalable colored k-mer index for sensitive pseudoalignment against hundreds of thousands of bacterial genomes. Bioinformatics 2023;39:i260–i269. ISSN 1367-4811. 10.1093/bioinformatics/btad233. 37387143 PMC10311346

[vbaf111-B3] Alipanahi B , Muggli MD, Jundi M et al Metagenome SNP calling via read-colored de Bruijn graphs. Bioinformatics 2021;36:5275–81. ISSN 1367-4803. 10.1093/bioinformatics/btaa081. 32049324 PMC8016496

[vbaf111-B4] Almodaresi F , Pandey P, Patro R. Rainbowfish: a succinct colored de Bruijn graph representation. bioRxiv, 10.1101/138016, 2017, preprint: not peer reviewed.

[vbaf111-B5] Almodaresi F , Pandey P, Ferdman M et al An efficient, scalable and exact representation of high-dimensional color information enabled via de Bruijn graph search. In: *Research in Computational Molecular Biology: 23rd Annual International Conference, RECOMB 2019, Washington, DC, USA, May 5-8, 2019, Proceedings 23*, pp.1–18. Springer, 2019.

[vbaf111-B6] Altschul SF , Gish W, Miller W et al Basic local alignment search tool. J Mol Biol 1990;215:403–10. 2231712 10.1016/S0022-2836(05)80360-2

[vbaf111-B7] Arakawa Y , Navarro G, Sadakane K. Bi-directional r-indexes. In: Bannai H and Holub J (eds), *33rd Annual Symposium on Combinatorial Pattern Matching (CPM 2022)*, *Dagstuhl, Germany*, 2022. Vol. 223 of *Leibniz International Proceedings in Informatics (LIPIcs)*, pp.11:1–11:14. Schloss Dagstuhl – Leibniz-Zentrum für Informatik. ISBN 978-3-95977-234-1. 10.4230/LIPIcs.CPM.2022.11.

[vbaf111-B8] Baire A , Marijon P, Andreace F et al Back to sequences: find the origin of k-mers. JOSS 2024;9:7066. 10.21105/joss.07066.

[vbaf111-B9] Bankevich A , Bzikadze AV, Kolmogorov M et al Multiplex de Bruijn graphs enable genome assembly from long, high-fidelity reads. Nat Biotechnol 2022;40:1075–81. 35228706 10.1038/s41587-022-01220-6

[vbaf111-B10] Bannai H , Gagie T, Tomohiro I. Refining the r-index. Theor Comput Sci 2020;812:96–108.

[vbaf111-B11] Benoit G , Lemaitre C, Lavenier D et al Reference-free compression of high throughput sequencing data with a probabilistic de Bruijn graph. BMC Bioinformatics 2015;16:1–14. 26370285 10.1186/s12859-015-0709-7 PMC4570262

[vbaf111-B12] Benoit G , Raguideau S, James R et al High-quality metagenome assembly from long accurate reads with metaMDBG. Nat Biotechnol 2024;42:1378–83. 38168989 10.1038/s41587-023-01983-6 PMC11392814

[vbaf111-B13] Bowe A , Onodera T, Sadakane K et al Succinct de Bruijn graphs. In: R Ben T Jijun (eds), Algorithms in Bioinformatics. Berlin, Heidelberg: Springer, 2012, 225–35. ISBN 978-3-642-33122-0.

[vbaf111-B14] Chikhi R , Limasset A, Jackman S et al On the representation of de Bruijn graphs. J Comput Biol 2015;22:336–52. 25629448 10.1089/cmb.2014.0160

[vbaf111-B15] Fan J , Khan J, Singh NP et al Fulgor: a fast and compact k-mer index for large-scale matching and color queries. Algorithms Mol Biol 2024;19:3. ISSN 1748-7188. 10.1186/s13015-024-00251-9. 38254124 PMC10810250

[vbaf111-B16] Groot Koerkamp R , Pibiri GE. The mod-minimizer: a simple and efficient sampling algorithm for long k-mers. In: Pissis SP and Sung W-K (eds), *24th International Workshop on Algorithms in Bioinformatics (WABI 2024)*, volume 312 of *Leibniz International Proceedings in Informatics (LIPIcs)*, pp.11:1–11:23, Dagstuhl, Germany, 2024. Schloss Dagstuhl – Leibniz-Zentrum für Informatik. ISBN 978-3-95977-340-9. 10.4230/LIPIcs.WABI.2024.

[vbaf111-B17] Guidi G , Ellis M, Rokhsar D et al *BELLA: Berkeley Efficient Long-Read to Long-Read Aligner and Overlapper*, pages 123–134. 10.1137/1.9781611976830.12.

[vbaf111-B18] He Y , Chu Y, Guo S et al T2t-yao: a telomere-to-telomere assembled diploid reference genome for Han Chinese. Genom Proteom Bioinform 2023;21:1085–100. 10.1016/j.gpb.2023.08.001 PMC11082261 37595788

[vbaf111-B19] Heinzl L , Hurdelhey B, Boissier M et al Evaluating lightweight integer compression algorithms in column-oriented in-memory DBMS. In: *ADMS@ VLDB*. 2021, 26–36.

[vbaf111-B20] Iqbal Z , Caccamo M, Turner I et al De novo assembly and genotyping of variants using colored de Bruijn graphs. Nat Genet 2012;44:226–32. 22231483 10.1038/ng.1028 PMC3272472

[vbaf111-B21] Karasikov M , Mustafa H, Rätsch G et al Lossless indexing with counting de Bruijn graphs. Genome Res 2022;32:1754–64. 35609994 10.1101/gr.276607.122 PMC9528980

[vbaf111-B22] Lee D , Song G. FastqCLS: a FASTQ compressor for long-read sequencing via read reordering using a novel scoring model. Bioinformatics 2022;38:351–6. 34623374 10.1093/bioinformatics/btab696

[vbaf111-B23] Li H. Fast construction of FM-index for long sequence reads. Bioinformatics 2014;30:3274–5. 25107872 10.1093/bioinformatics/btu541 PMC4221129

[vbaf111-B24] Limasset A , Flot J-F, Peterlongo P. Toward perfect reads: self-correction of short reads via mapping on de Bruijn graphs. Bioinformatics 2020;36:1374–81. 30785192 10.1093/bioinformatics/btz102

[vbaf111-B25] Limasset A , Rizk G, Chikhi R et al Fast and scalable minimal perfect hashing for massive key sets. In: Costas SI, Solon PP, Simon JP *et al.* (eds), *16th International Symposium on Experimental Algorithms (SEA 2017)*, volume 75 of *Leibniz International Proceedings in Informatics (LIPIcs)*, pages 25:1–25:16, Dagstuhl, Germany, 2017. Schloss Dagstuhl – Leibniz-Zentrum für Informatik. ISBN 978-3-95977-036-1. 10.4230/LIPIcs.SEA.2017.25.

[vbaf111-B26] Liu B , Guo H, Brudno M et al deBGA: read alignment with de Bruijn graph-based seed and extension. Bioinformatics 2016;32:3224–32. 27378303 10.1093/bioinformatics/btw371

[vbaf111-B27] Lyman CA , Fujimoto MS, Suvorov A et al Whole genome phylogenetic tree reconstruction using colored de Bruijn graphs. In: *2017 IEEE 17th International Conference on Bioinformatics and Bioengineering (BIBE)*, Washington, DC, USA. 2017, 260–65. 10.1109/BIBE.2017.00-44.

[vbaf111-B28] Marchet C , Limasset A. Scalable sequence database search using partitioned aggregated bloom comb trees. Bioinformatics 2023;39:i252–i259. 37387170 10.1093/bioinformatics/btad225 PMC10311332

[vbaf111-B29] Marchet C , Boucher C, Puglisi SJ et al Data structures based on k-mers for querying large collections of sequencing data sets. Genome Res 2021a;31:1–12. 33328168 10.1101/gr.260604.119 PMC7849385

[vbaf111-B30] Marchet C , Kerbiriou M, Limasset A. Blight: efficient exact associative structure for k-mers. Bioinformatics 2021b;37:2858–65. 33821954 10.1093/bioinformatics/btab217

[vbaf111-B31] Marchet C , Lecompte L, Limasset A et al A resource-frugal probabilistic dictionary and applications in bioinformatics. Discret Appl Math 2020;274:92–102.

[vbaf111-B32] Nagarajan N , Pop M. Parametric complexity of sequence assembly: theory and applications to next generation sequencing. J Comput Biol 2009;16:897–908. 19580519 10.1089/cmb.2009.0005

[vbaf111-B33] Nishimoto T , Tabei Y. Optimal-time queries on BWT-runs compressed indexes. In: Bansal N, Merelli E, and Worrell J (eds), *48th International Colloquium on Automata, Languages, and Programming (ICALP 2021)*, volume 198 of *Leibniz International Proceedings in Informatics (LIPIcs)*, pages 101:1–101:15, Dagstuhl, Germany, 2021. Schloss Dagstuhl – Leibniz-Zentrum für Informatik. ISBN 978-3-95977-195-5. 10.4230/LIPIcs.ICALP.2021.101.

[vbaf111-B34] Nurk S , Koren S, Rhie A et al The complete sequence of a human genome. Science 2022;376:44–53. 35357919 10.1126/science.abj6987 PMC9186530

[vbaf111-B35] Pellow D , Pu L, Ekim B et al Efficient minimizer orders for large values of k using minimum decycling sets. Genome Res 2023;33:1154–61. 37558282 10.1101/gr.277644.123 PMC10538483

[vbaf111-B36] Pibiri GE. Sparse and skew hashing of k-mers. Bioinformatics 2022;38:i185–i194. 35758794 10.1093/bioinformatics/btac245 PMC9235479

[vbaf111-B37] Pibiri GE , Shibuya Y, Limasset A. Locality-preserving minimal perfect hashing of k-mers. Bioinformatics 2023;39:i534–i543. 37387137 10.1093/bioinformatics/btad219 PMC10311298

[vbaf111-B38] Pibiri GE , Trani R. Pthash: revisiting FCH minimal perfect hashing. *CoRR*, abs/2104.10402, https://arxiv.org/abs/2104.10402, 2021, preprint: not peer reviewed.

[vbaf111-B39] Ramos LP , Louza FA, Telles GP. Comparative genomics with succinct colored de Bruijn graphs. arxiv, https://arxiv.org/abs/2411.09114, 2024, preprint: not peer reviewed.

[vbaf111-B40] Roberts M , Hayes W, Hunt BR et al Reducing storage requirements for biological sequence comparison. Bioinformatics 2004;20:3363–9. 15256412 10.1093/bioinformatics/bth408

[vbaf111-B41] Rossi M , Oliva M, Langmead B et al Moni: a pangenomic index for finding maximal exact matches. J Comput Biol 2022;29:169–87. 35041495 10.1089/cmb.2021.0290 PMC8892979

[vbaf111-B42] Solomon B , Kingsford C. Improved search of large transcriptomic sequencing databases using split sequence bloom trees. J Comput Biol 2018;25:755–65. 29641248 10.1089/cmb.2017.0265 PMC6067102

[vbaf111-B43] Trotman A , Lin J. In vacuo and in situ evaluation of SIMD Codecs. In: *Proceedings of the 21st Australasian Document Computing Symposium (ADCS '16)*. New York, NY, USA: Association for Computing Machinery, 2016, 1–8. 10.1145/3015022.3015023.

[vbaf111-B44] Wang M , Ye Y, Tang H. A de Bruijn graph approach to the quantification of closely-related genomes in a microbial community. J Comput Biol 2012;19:814–25. 22697249 10.1089/cmb.2012.0058 PMC3375647

[vbaf111-B45] Xiao C-L , Chen Y, Xie S-Q et al MECAT: fast mapping, error correction, and de novo assembly for single-molecule sequencing reads. Nat Methods 2017;14:1072–4. 28945707 10.1038/nmeth.4432

[vbaf111-B46] Zakeri M , Brown NK, Ahmed OY et al Movi: a fast and cache-efficient full-text pangenome index. iScience 2024;27:111464. ISSN 2589-0042. 10.1016/j.isci.2024.111464 . 39758981 PMC11696632

[vbaf111-B47] Zheng H , Kingsford Carl, Marçais G. Improved design and analysis of practical minimizers. Bioinformatics 2020;36:i119–i127. 32657376 10.1093/bioinformatics/btaa472 PMC8248892

