# Supplementary material for: K2R: Tinted de Bruijn graphs implementation for efficient read extraction from sequencing datasets
Source: Bioinform Adv. 2025 May 14;5(1):vbaf111. doi: 10.1093/bioadv/vbaf111 (PMC12257931; doi:10.1093/bioadv/vbaf111)
Supplement: vbaf111_Supplementary_Data [file vbaf111_supplementary_data.zip › K2R_appendix.pdf]

Appendix

Contents

1 Implementation Details **2**
1.1 Index Structure 2
1.2 Minimizer Abundance Filtering 2
1.3 Parallelization Overview 2
1.4 Minimizer Scheme 2
1.5 Homocompression 2

2 Parameter influence **4**
2.1 Counting Bloom Filter and minimizer size 4
Figure S1 : Ratio between verified and kept reads, querying datasets with different minimizer sizes. 4
2.2 Error rate 4
Figure S2 : Ratio between verified and kept reads, querying datasets with two different error rates. 5

3 Supplementary Figures **5**
Figure S3 : Comparison of ressource usage for simulated reads 6
Figure S5 : Comparison of structure sizes for simulated reads depending on coverage 8
Figure S6 : Comparison of structure sizes for simulated reads depending on error rates 9
Figure S7 : Comparison of ressource usage for several *E.Coli* datasets using several threads 10
Figure S7 : Comparison of ressource usage for several *C.Elegans* datasets using several threads 11
Figure S9 : Comparison of ressource usage during "positive" queries 12
Figure S10 : Comparison of ressource usage during "random" queries 13
Figure S11 : Global use of K2R 14

1 Implementation Details

1.1 Index Structure

In our index structure, we employ a dual mapping system consisting of a [minimizer: color identifier] map and an [color identifier: color] map, where minimizers and color identifiers are integers, and colors are an object composed of a compressed integer array, an integer array and several integers (representing structure sizes and occurrencies, for implementation purpose). We opted to use the unordered_dense hash map, available at `http://github.com/martinus/unordered_dense`, due to its exceptional performance characteristics, which have been leveraged in several bioinformatics tools, including kallisto [2] and mashmap [3].

Upon inserting a read into the index, its minimizers are computed and inserted into the minimizer map. Each corresponding identifier is then updated to incorporate the new read identifier into its color list. If the associated color does not preexist within the map, a new identifier is generated and added to the identifier map, along with its newly associated color. Colors are compressed using the TurboPFor library `http://github.com/powturbo/TurboPFor-Integer-Compression`, which displays almost state-of-the-art compression levels at a very low computational cost.

To keep the index lean and efficient, the identifier map also tracks the number of minimizers referencing each identifier. When a minimizer's association shifts to a different identifier, two scenarios arise. First, if one or more minimizers is still associated to the former color identifier, if the new color exists, the minimizer can be automatically associated to it, otherwise a new color is created using the former one. Second, if the color is no more associated to any minimizer, this color is not deleted but modified to correspond to its last minimizers, adding a read identifier.

Our choice of index structure imbues it with dynamic capabilities, allowing for real-time querying at any point during the read insertion process. While static structures may offer advantages in memory efficiency, they lack flexibility and require a more cumbersome construction phase.

1.2 Minimizer Abundance Filtering

To enhance construction efficiency and minimize the index size, we implement an optional filtering mechanism for weak minimizers using a Counting Bloom filter to approximate minimizer abundance within the dataset. It's important to note that a Counting Bloom filter can only overestimate counts, leading to false positives by misclassifying some weak minimizers as solid due to hash collisions with genuinely solid minimizers.

After approximate minimizer counting, we convert the Counting Bloom filter into a Regular Bloom filter by transforming each cell, originally representing counts with integers, into a single bit. This bit indicates the solidity of the associated minimizer, significantly reducing memory consumption during the actual index construction. Using this strategy, we limit the peak memory usage as the hash tables should become the memory bottleneck using a well sized Bloom filter.

We also observe the opposite pattern of overabundant minimizers linked to highly repeated regions in a genome, either due to biological mechanisms or sequencing type. Since such minimizers can be associated with an incredibly high number of reads, they can seriously hinder index performance and "pollute" the output with many irrelevant matches. To counteract this, we implement an optional maximum minimizer abundance filtering, as commonly performed by other tools [4].

When employing minimizers in place of k-mers, alongside Counting Bloom filters to sift through them, a natural concern arises regarding the potential influence of false positives introduced by this method on the final output. To address this, we conduct an evaluation illustrated in Figure S1, where we calculate the proportion of reads selected based on their minimizer content relative to the total number of reads achieving the requisite k-mer similarity threshold (here fixed at 0.3). Remarkably, a substantial majority of queries exhibit a ratio exceeding 75%, indicating that a majority of the selected reads are, in fact, akin to the query. This high ratio underscores the efficiency of our approach in maintaining a strong correlation between selected reads and their relevance to the query.

1.3 Parallelization Overview

The filtering phase is parallelized with a mutex array to secure sections of the Counting Bloom Filter, enabling efficient concurrent operations. Parallelizing the index construction phase is more complex due to dependencies on synchronized maps. We mitigate this by implementing an inter-reads parallelization, where minimizers are computed across sequence substrings concurrently. Tasks related to color management, including decompression, updates, sort and recompression, are executed in parallel, protected by mutex arrays to ensure data integrity. This setup allows concurrent modifications to the color and m-mer maps without data corruption.

The queries can also be executed in parallel, with each thread handling a single query.

1.4 Minimizer Scheme

Minimizers reduce complexity by representing a set of overlapping k-mers with a single m-mer. The ideal minimizer selection scheme would identify one m-mer for every $k-m+1$ sequence of overlapping k-mers. Practical implementations, such as the random minimizer strategy, tend to select approximately twice as many minimizers as the ideal case.

Recent advancements in minimizer selection techniques aim to closely approach this theoretical lower bound, thus reducing the quantity of necessary minimizers. We employ decycling set minimizers [5], which minimize the count of selected minimizers, albeit at the cost of increased computational overhead.

1.5 Homocompression

To address homopolymer run length errors prevalent in HiFi and PacBio sequencing reads, we propose an optional 'homocompression' feature. This technique lossily represents sequences within reads by compressing consecutive occurrences of a nucleotide X...X into a single instance of X. As highlighted in the La Jolla Assembler paper [1], applying homocompression to HiFi reads can result in a threefold reduction in total error count, making a substantial proportion of reads error-free. This approach not only improves sequencing data accuracy but also enhances the overall reliability of genomic analysis by significantly reducing the impact of homopolymer-associated errors.

References

[1] Anton Bankevich, Andrey V Bzikadze, Mikhail Kolmogorov, Dmitry Antipov, and Pavel A Pevzner. Multiplex de bruijn graphs enable genome assembly from long, high-fidelity reads. *Nature biotechnology*, 40(7):1075–1081, 2022.

[2] Nicolas L Bray, Harold Pimentel, Páll Melsted, and Lior Pachter. Near-optimal probabilistic rna-seq quantification. *Nature biotechnology*, 34(5):525–527, 2016.

[3] Chirag Jain, Alexander Dilthey, Sergey Koren, Srinivas Aluru, and Adam M Phillippy. A fast approximate algorithm for mapping long reads to large reference databases. In *International Conference on Research in Computational Molecular Biology*, pages 66–81. Springer, 2017.

[4] Heng Li. Minimap2: pairwise alignment for nucleotide sequences. *Bioinformatics*, 34(18):3094–3100, 2018.

[5] David Pellow, Lianrong Pu, Bariş Ekim, Lior Kotlar, Bonnie Berger, Ron Shamir, and Yaron Orenstein. Efficient minimizer orders for large values of k using minimum decycling sets. *Genome Research*, 33(7):1154–1161, 2023.

2 Parameter influence

2.1 Counting Bloom Filter and minimizer size

The size of the counting Bloom filter and the minimizers' will influence the number of collisions: the smaller the filter and the minimizers are, the higher the number of collisions is, leading to an increase in false positives (see Figure S1). Conversely, a larger filter or minimizer will reduce collisions but require more memory. It is worth noting that, in the case of K2R, this filter is the most memory-intensive component.

However, there is a subtlety: for a very small filter size (compared to the size of the dataset to be indexed), the number of k-mers sharing the same hash value increases as the number of available values decreases. After an initial rise in the false positive rate, if the filter size becomes too small, this rate eventually decreases because the majority of the selected k-mers will be correct.

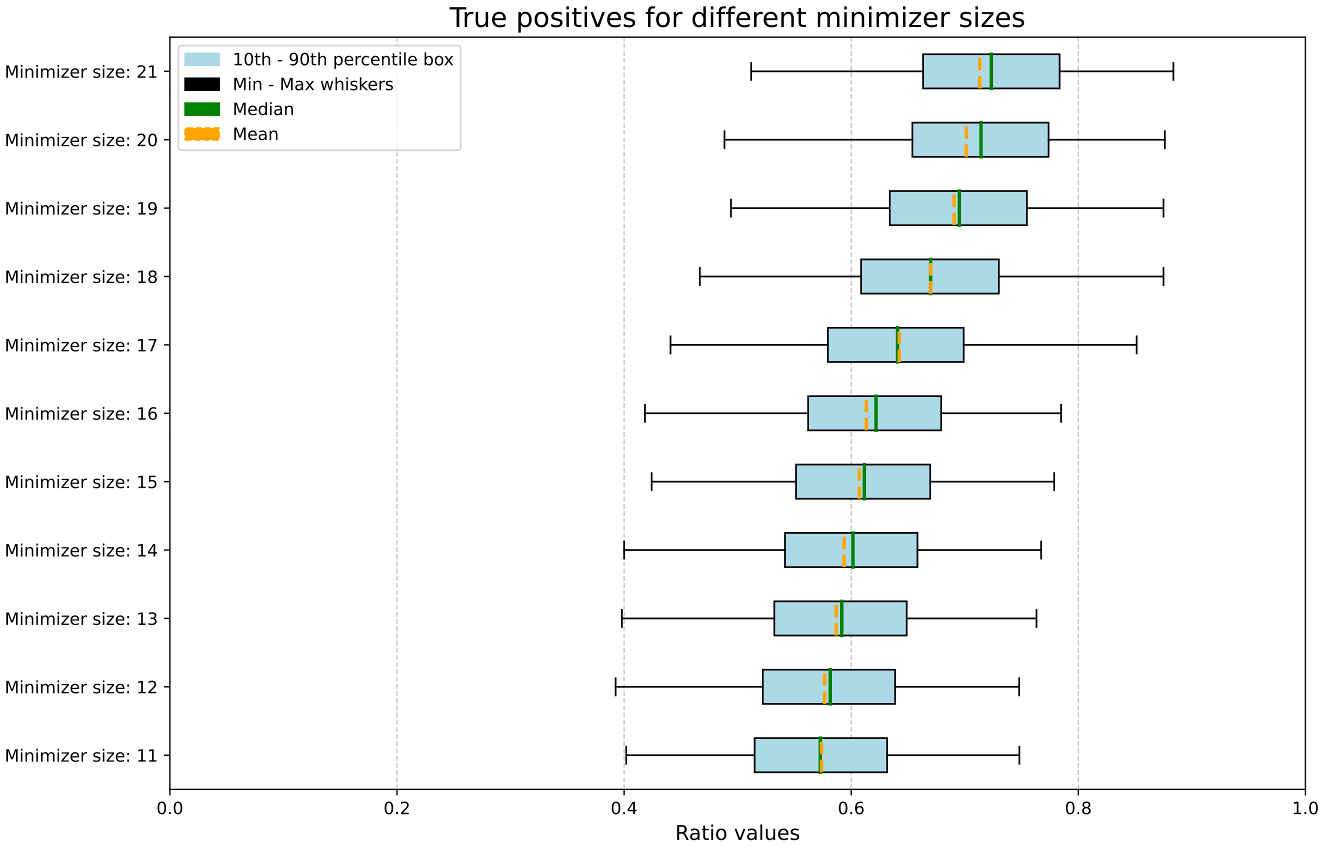

Figure S1: Ratio between verified and kept reads, during queries of 10,000 sequences of length 10,000 without any errors using K2R with a threshold of 0.6. The index is created from simulated reads from the *E.Coli* genome of length 10,000, with a 200X coverage, an error rate of 1%. The indexation is performed using different minimizer sizes, varying from 11 to 21.

2.2 Error rate

A high error rate will generate k-mers that are not necessarily present in the original file. In the case of queries, these slight differences will cause some k-mers, when manually verified, to ultimately be absent, thereby increasing the false positive rate (see Figure S2).

a

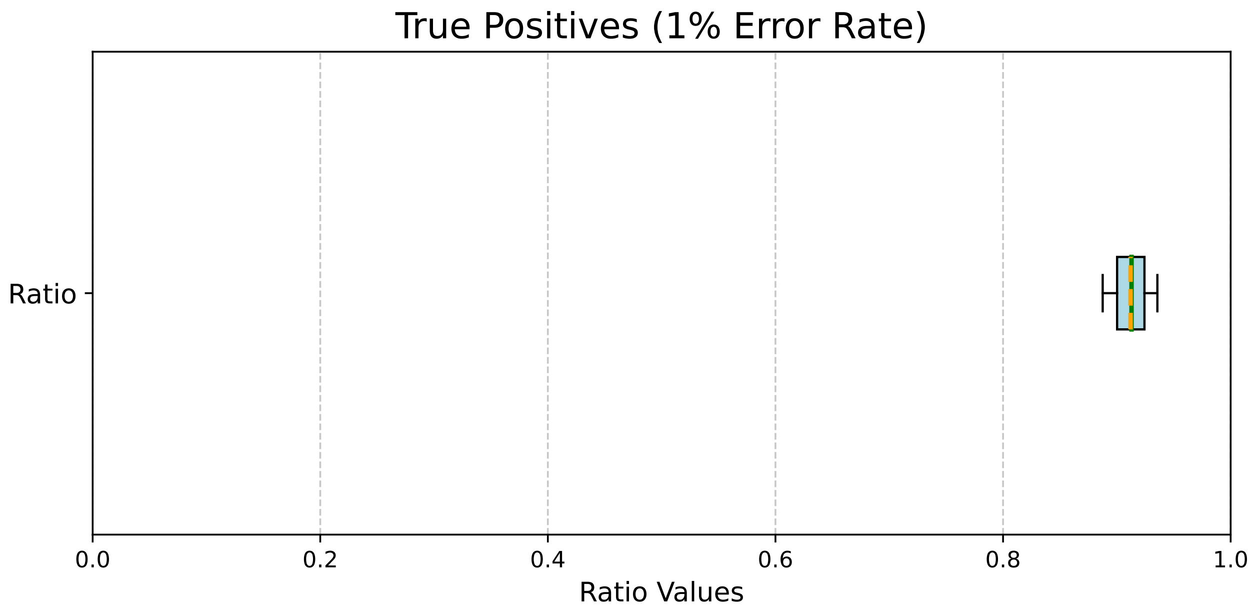

b

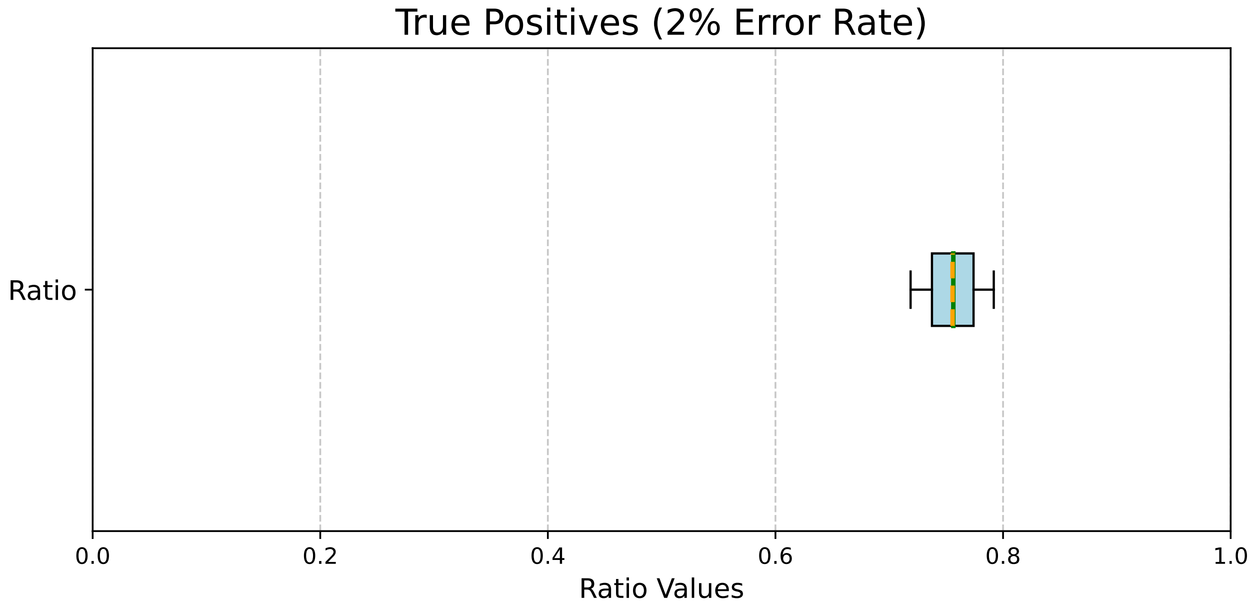

Figure S2: Ratio between verified and kept reads, during queries of 10,000 sequences of length 10,000 without any errors using K2R with a threshold of 0.3. The index is created from simulated reads from the *E.Coli* genome of length 10,000, with a 200X coverage and an error rate of 1% in (a) and 2% in (b).

3 Supplementary Figures

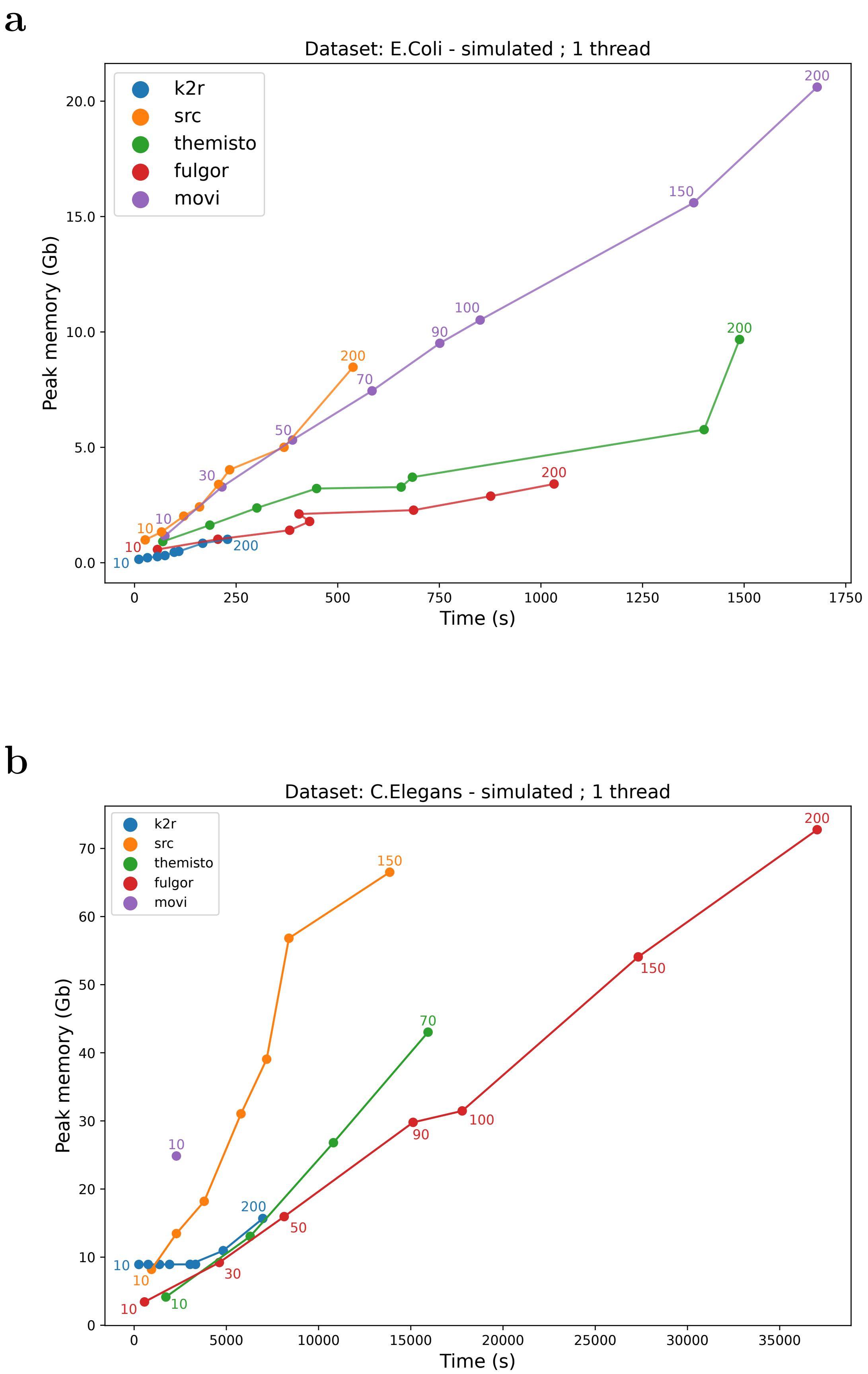

Figure S3: Comparison of ressource usage (wall-clock time and memory peak) used during the index construction with K2R, SRC, Movi, Fulgor and Themisto according to the available coverages. The dataset used consists of simulated reads from (a) *E.Coli* and (b) *C.Elegans* genomes, each with a length of 10,000 and a 1% error rate.

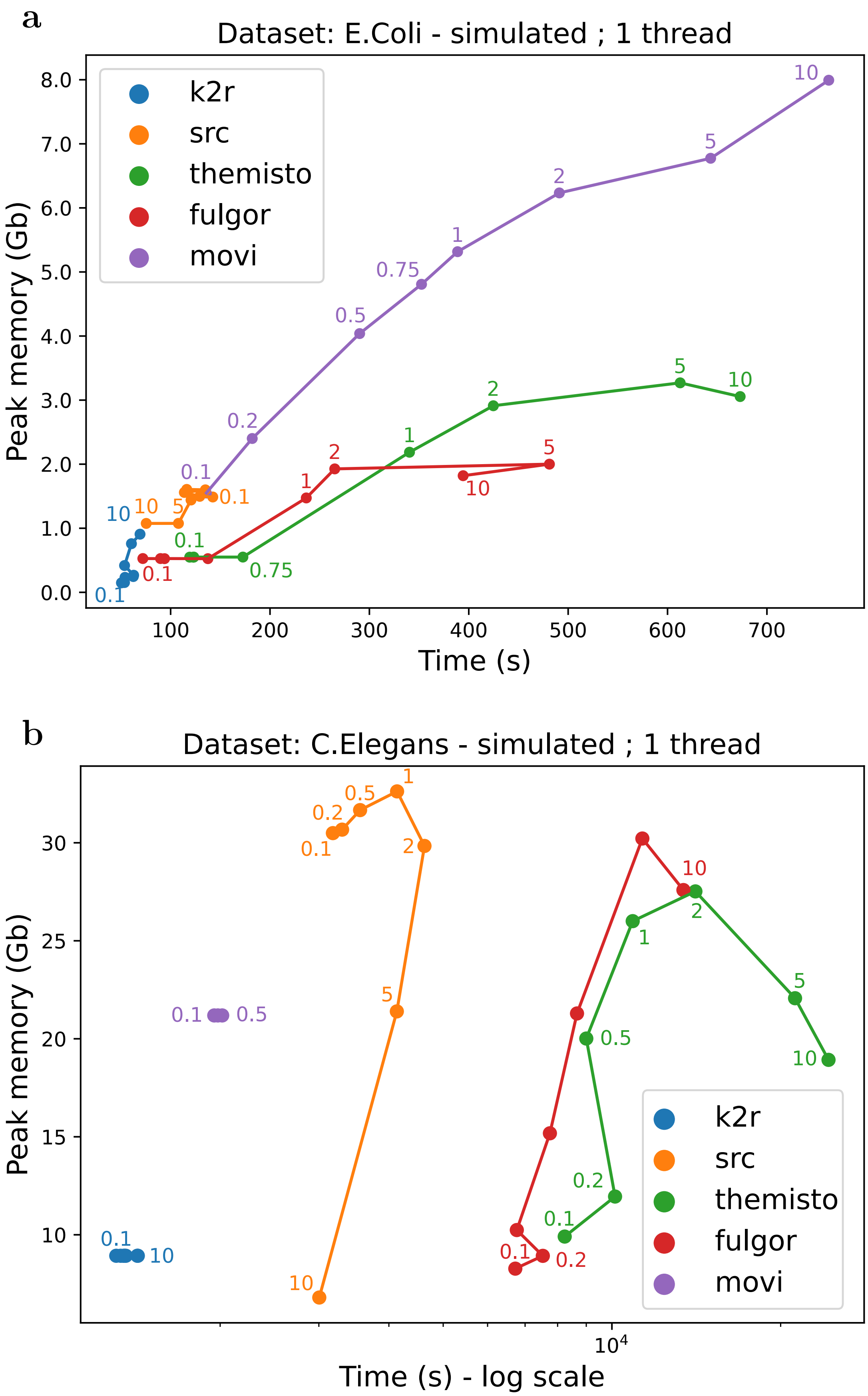

Figure S4: Memory peak and wall-clock time used during the index construction according to the error rate (specified on the labels in percent) for simulated reads (length of 10,000 with a 50X coverage) from (a) *E.Coli* and (b) *C.Elegans* genomes. This last graph is in logarithmic scale for readability purposes.

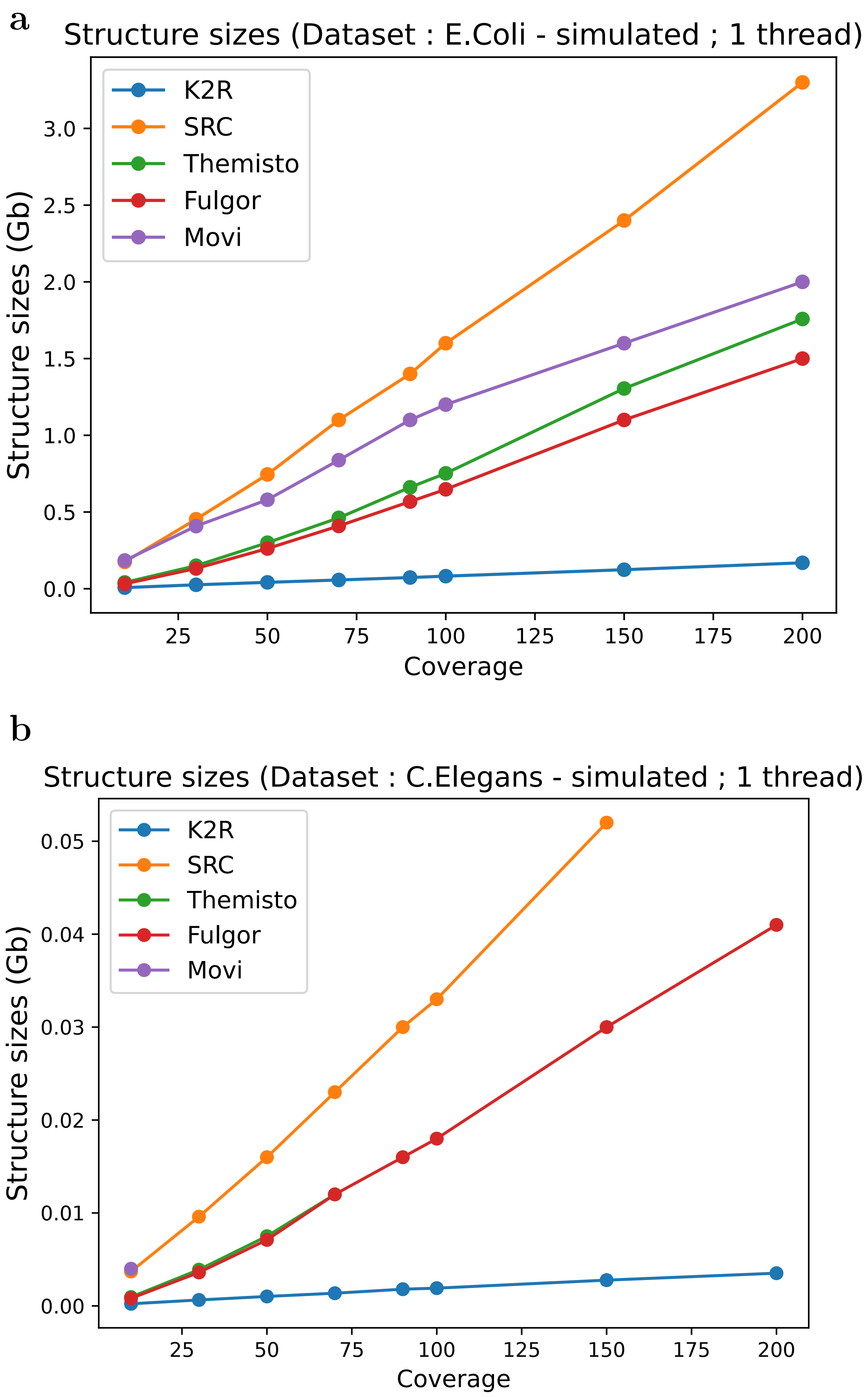

Figure S5: Comparison of the index size for different coverage values using simulated reads from (a) *E.Coli* and (b) *C.Elegans* reference genomes, with a 1% error rate and a read length of 10,000.

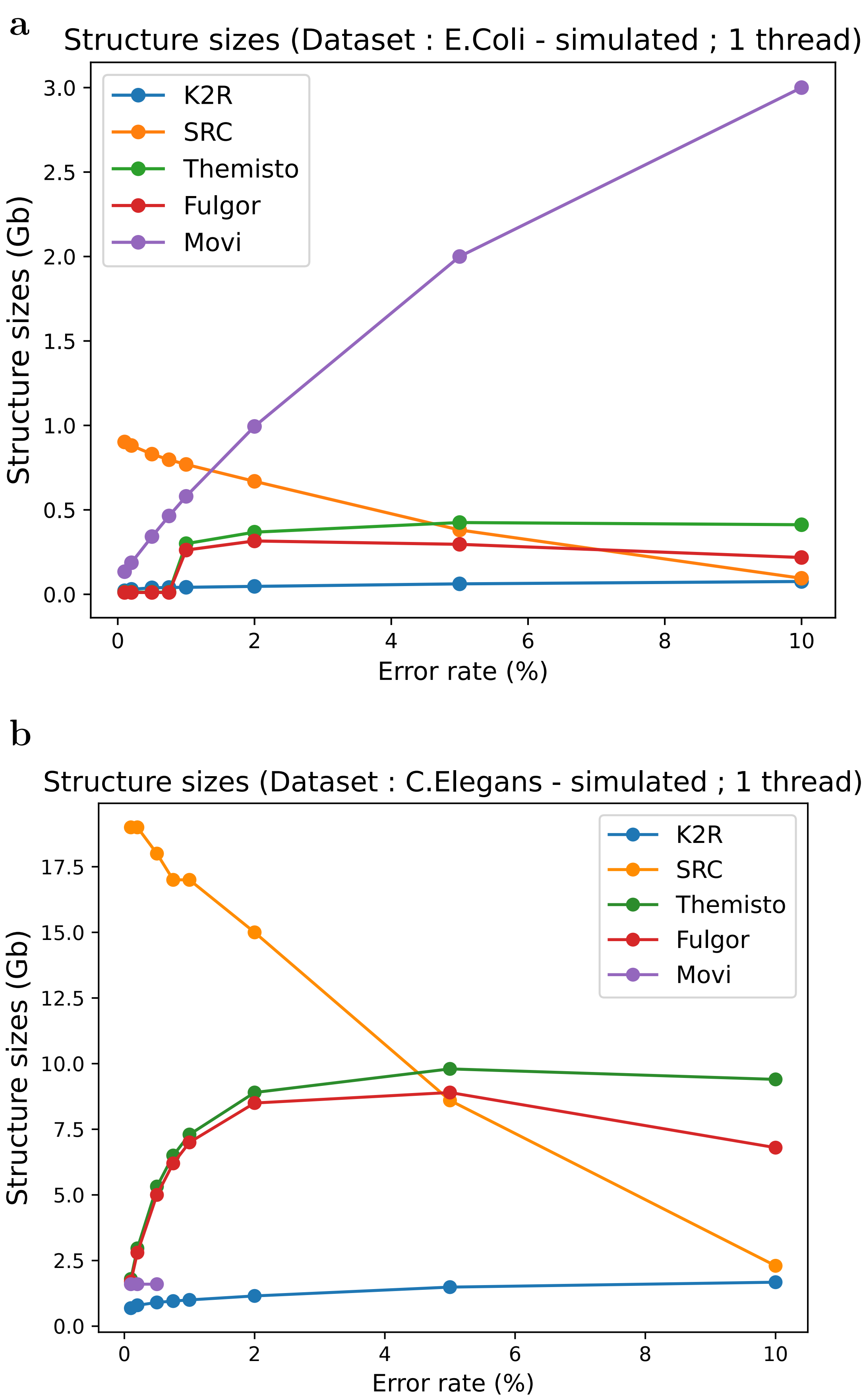

Figure S6: Comparison of the index size for different error rate values using simulated reads from (a) *E.Coli* and (b) *C.Elegans* reference genomes, with a coverage of 50X and a read length of 10,000.

a

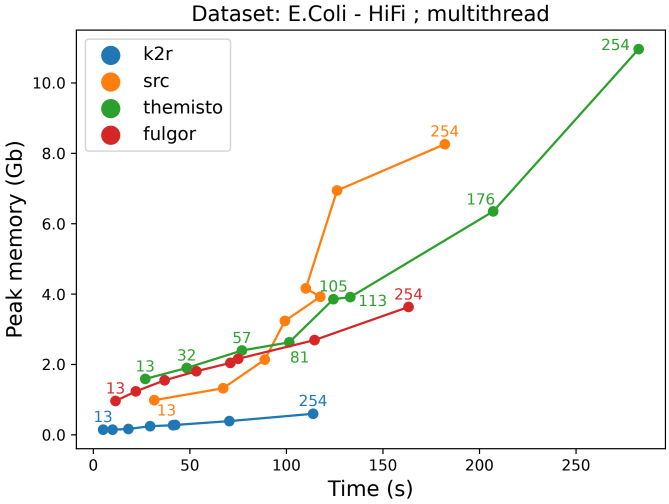

b

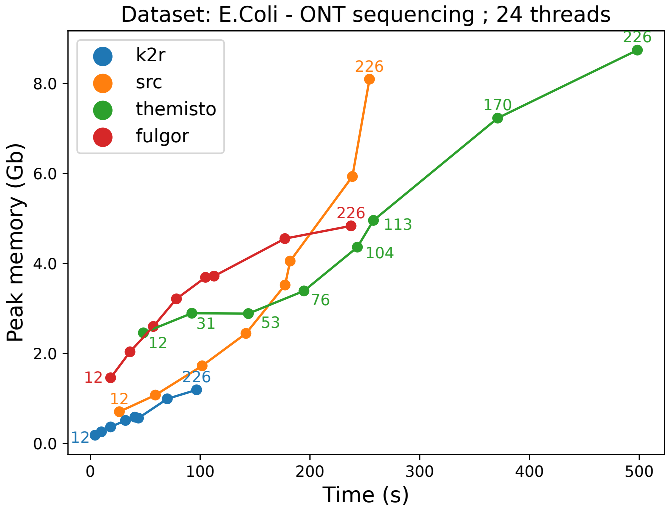

c

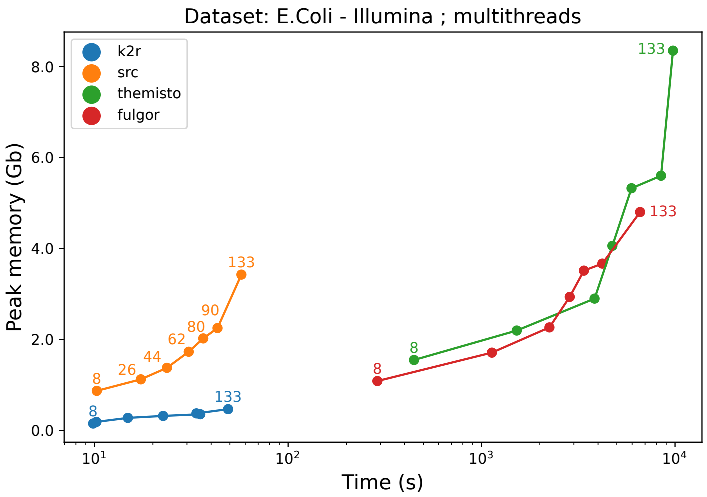

Figure S7: Comparison of ressource usage (wall-clock time and memory peak) used during the index construction with K2R, SRC, Fulgor and Themisto according to the available coverages, with several threads. The dataset used consists of reads from 3 different datasets of *E.Coli* genome : (a) HiFi dataset, (b) ONT dataset and (c) Illumina dataset, with different coverages.

a

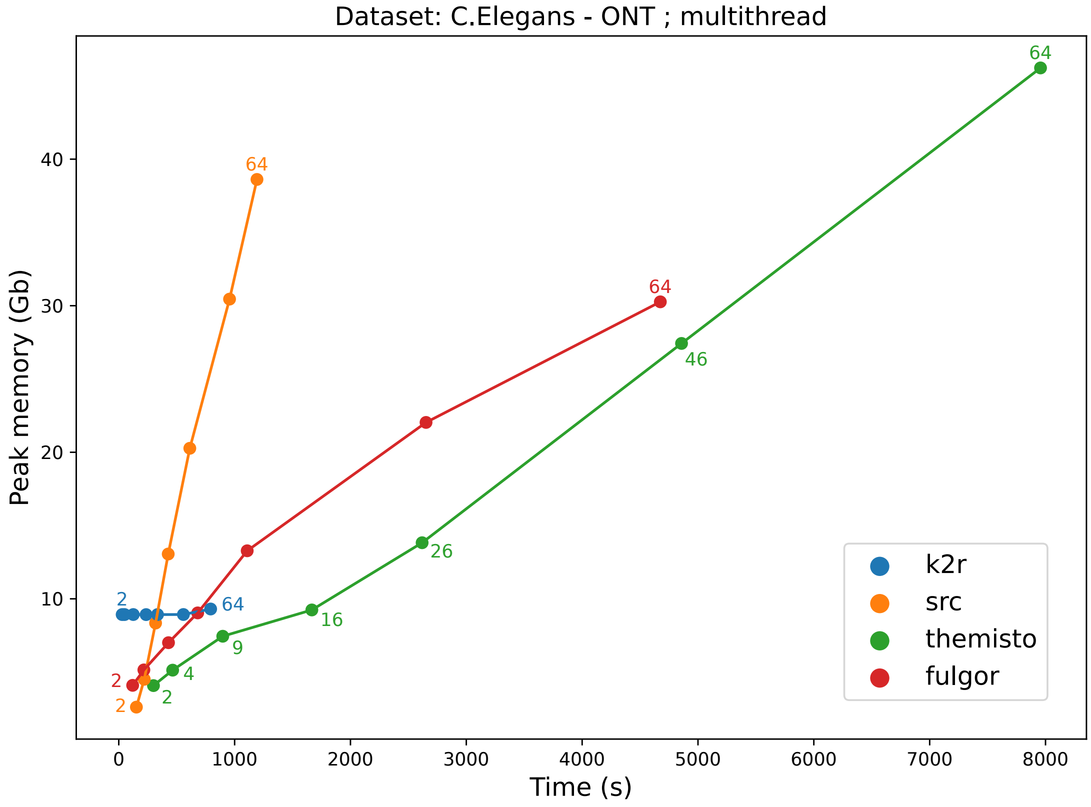

b

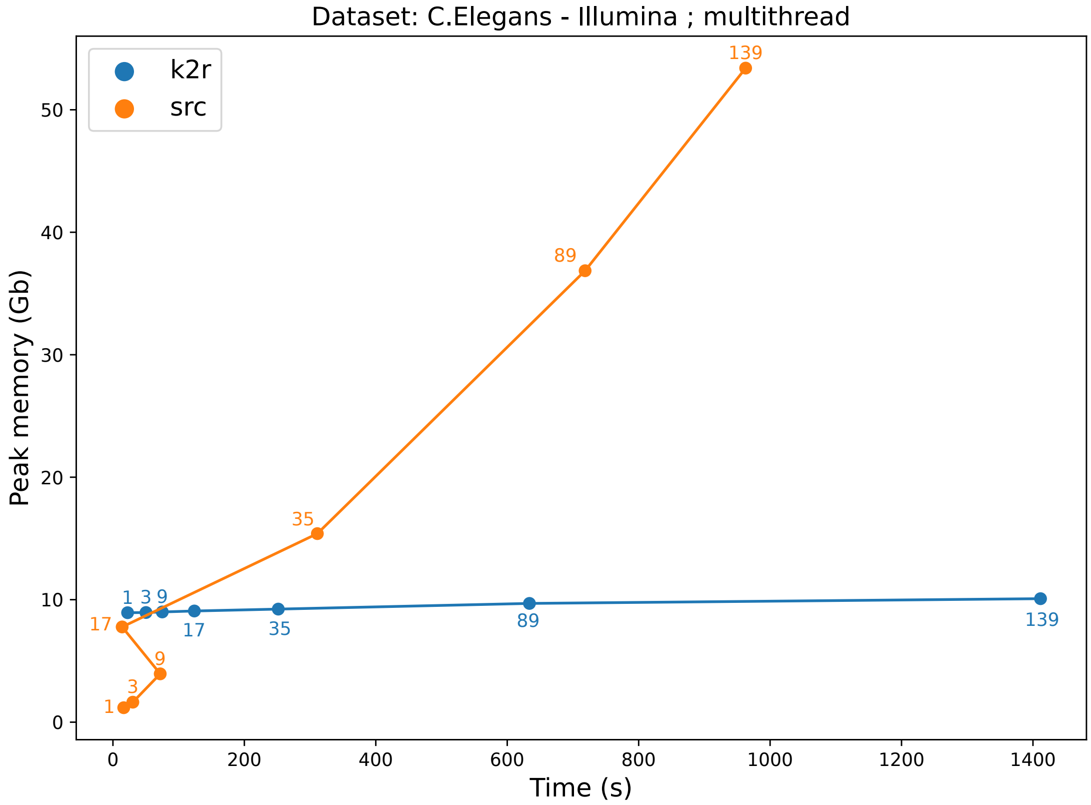

Figure S8: Comparison of ressource usage (wall-clock time and memory peak) used during the index construction with K2R and SRC, according to the available coverages, with several threads. The datasets used consists of reads from 2 different datasets of *C.Elegans* genome : (a) ONT dataset and (b) Illumina dataset, with different coverages. We notice that Themisto, Fulgor and Movi can't be tested here, because of their mode of use and scale up problems respectively.

a

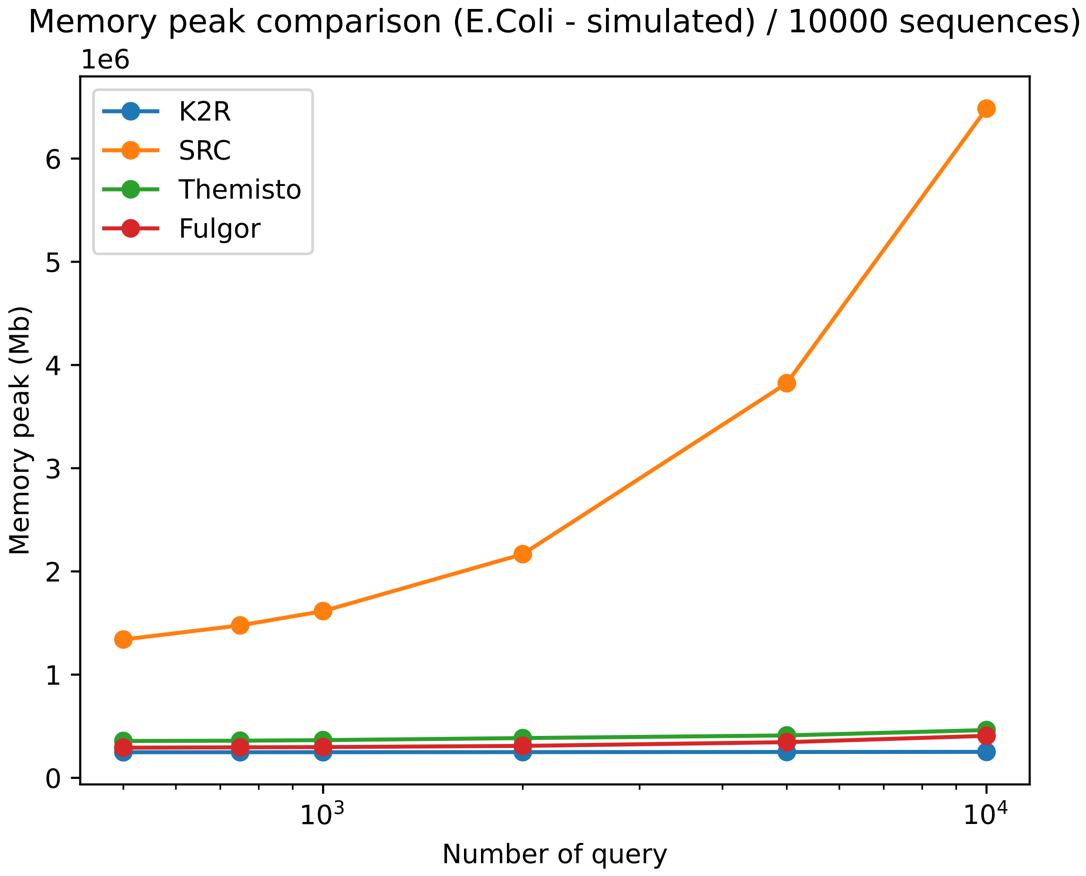

b

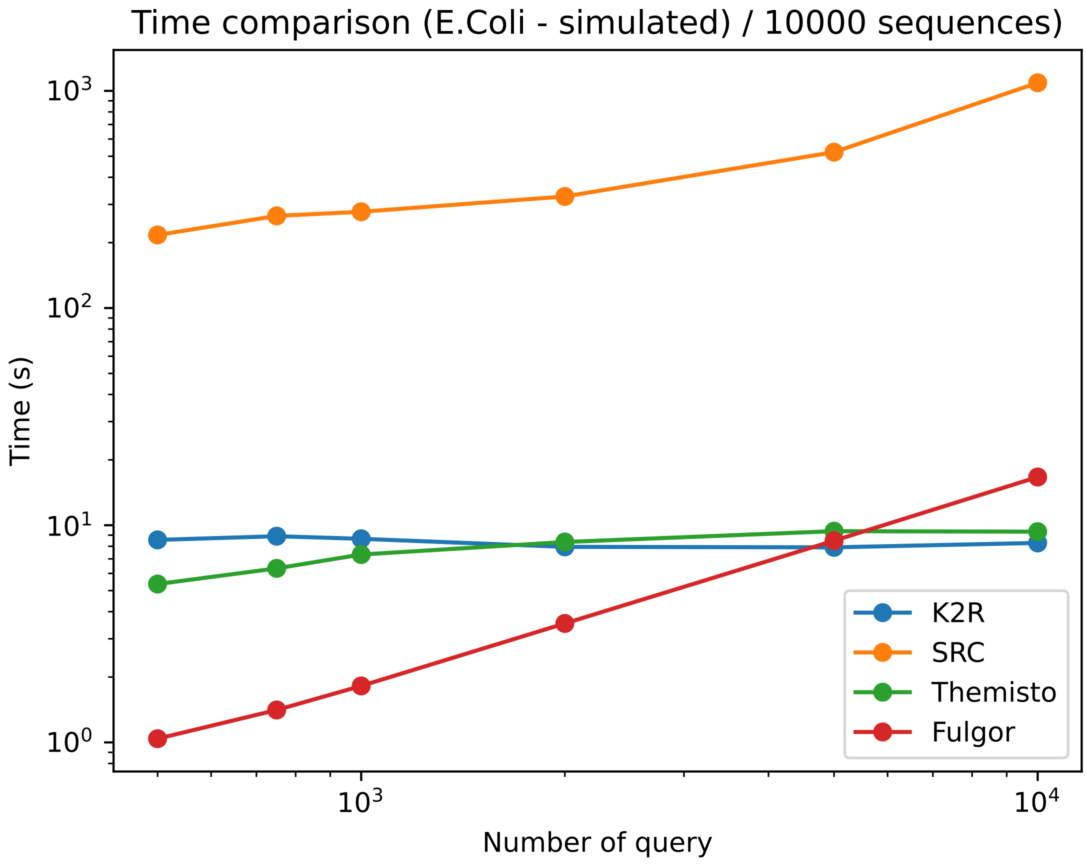

Figure S9: Comparison of the memory peak (a) and time consuming (b) during "positive" queries with K2R, SRC, Fulgor and Themisto according to the number of queried sequences. The sequences queried consists of 10,000 reads of length 10,000 from a simulated *E.Coli* dataset, having a 50X coverage and a 1% error rate.

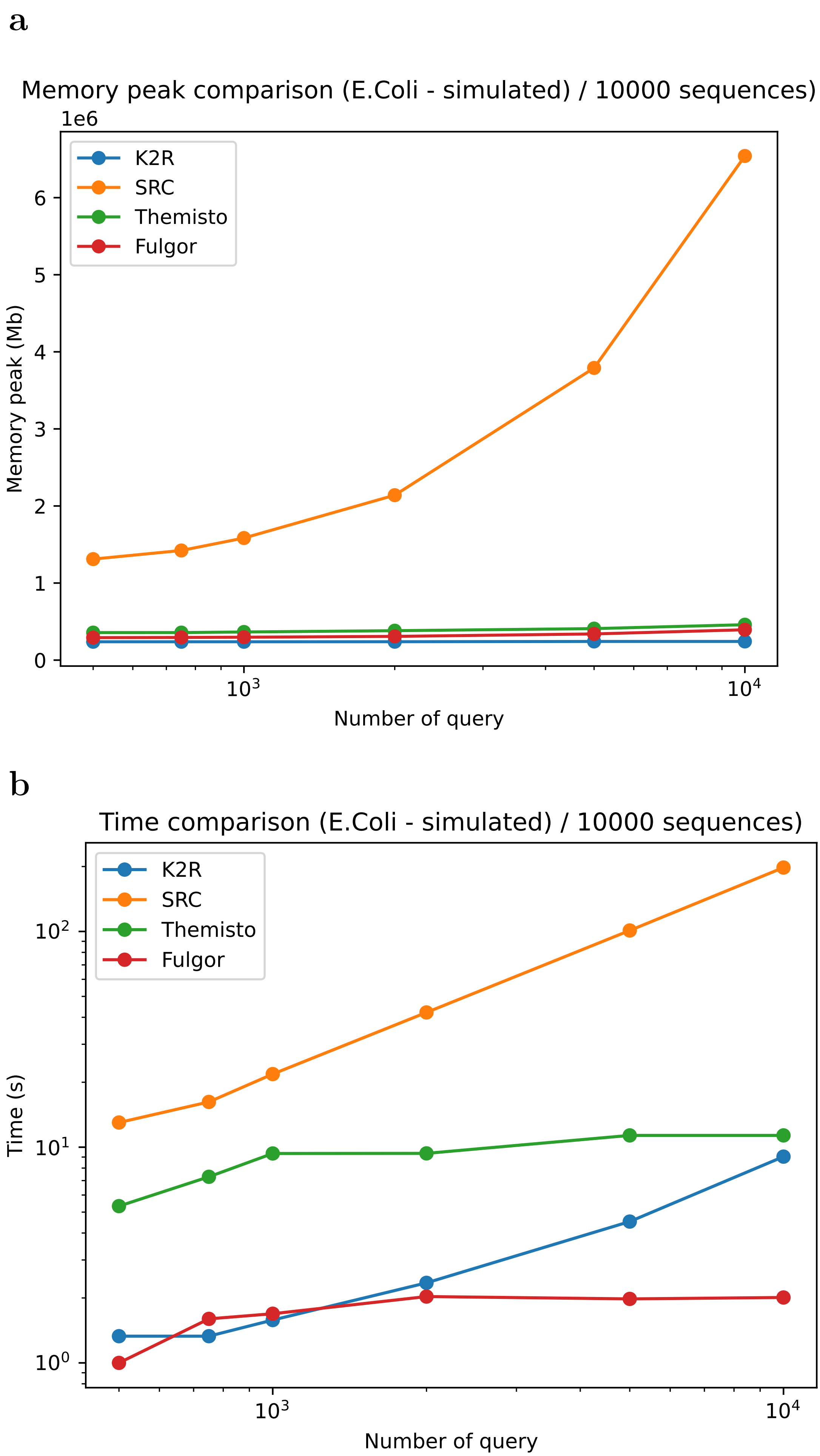

Figure S10: Comparison of the memory peak (a) and time consuming (b) during "negative" queries with K2R, SRC, Fulgor and Themisto according to the number of queried sequences. The sequences queried consists of 10,000 reads of length 10,000 from a simulated *E.Coli* dataset, having a 50X coverage and a 1% error rate.

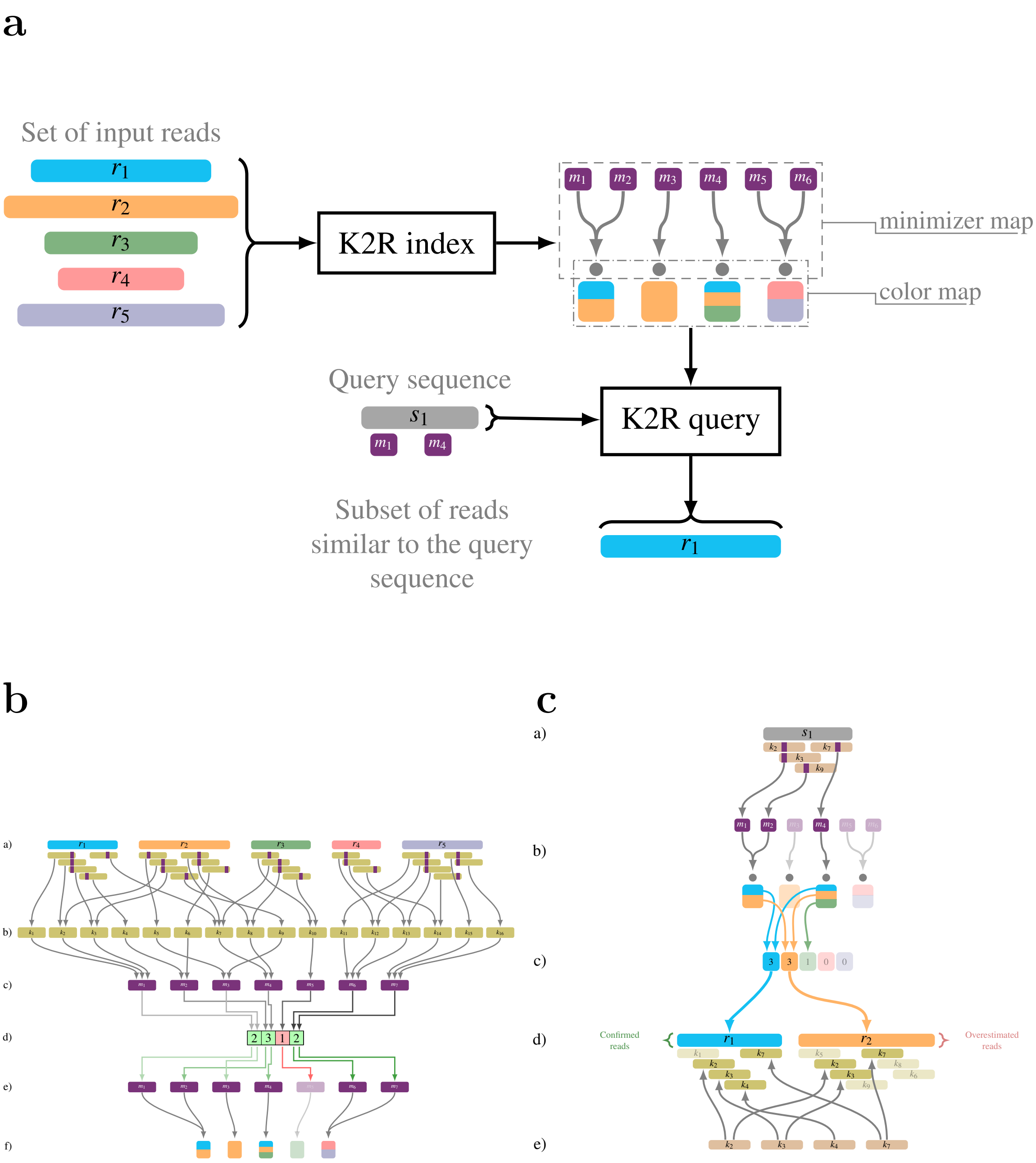

Figure S11: (a) Global use case of K2R: for finding the subset of reads similar to a sequence, we first use K2R index to index all the available reads. In a second step, we use K2R query on the sequence to find similar reads by using the index. (b) K2R index: For a set of input reads, we can extract the set of k-mers. Each k-mer is linked to a minimizer with possible collisions. By using counting filter, we can remove weak minimizer. Each remaining minimizer is linked to a color. We store this link from minimizers to colors by two maps : the minimizer map and the color map. (c) K2R query: For a query sequence, we extract the set of k-mers and minimizers. We use each minimizer of the query sequence in the index constructed by K2R index to compute the counting table of the reads indexed to select potential similar reads. Each k-mer of the query sequence is searched for in each of the potential similar reads to remove overestimated reads.
